# Supplementary material for: Proline Metabolism Genes in Transgenic Plants: Meta-Analysis under Drought and Salt Stress
Source: Plants (Basel). 2024 Jul 11;13(14):1913. doi: 10.3390/plants13141913 (PMC11280441; doi:10.3390/plants13141913)

Moderation analysis under stress conditions

Figure S1. Proline moderators

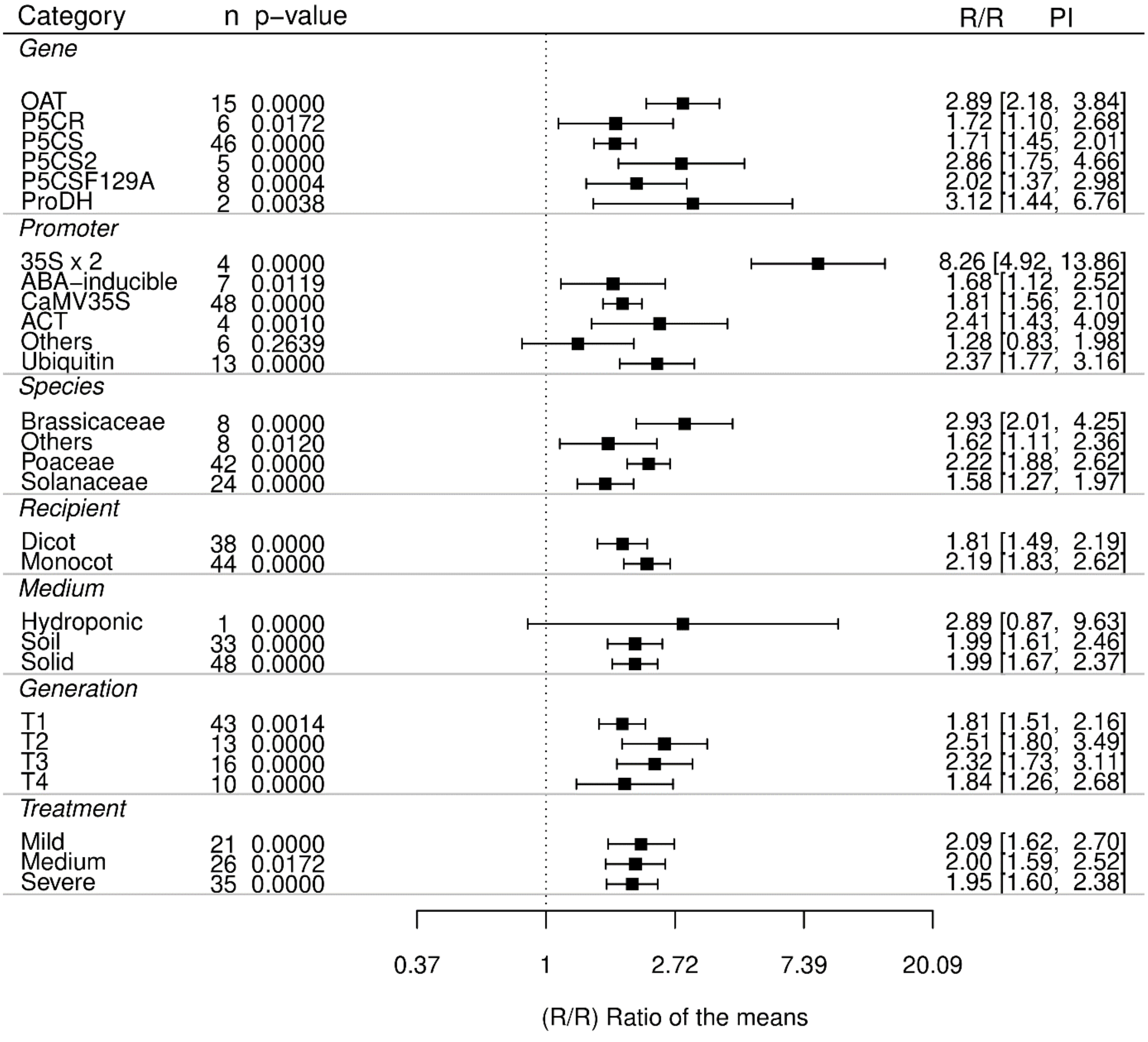

Figure S2. Plant height moderators

Category	n	p–value	R/R	PI
Gene				
OAT	6	0.0000	1.54	[1.38, 1.72]
Others	3	0.0000	1.63	[1.32, 2.00]
P5CS	7	0.0007	1.22	[1.09, 1.38]
Promoter				
35S x 2	4	0.0000	1.65	[1.46, 1.87]
CaMV35S	11	0.0000	1.29	[1.17, 1.42]
Ubiquitin	1	0.0296	1.44	[1.04, 1.99]
Species				
Fabaceae	3	0.0003	1.41	[1.17, 1.70]
Poaceae	12	0.0000	1.36	[1.24, 1.49]
Solanaceae	1	0.0000	2.25	[1.64, 3.09]
Recipient				
Dicot	4	0.0000	1.59	[1.35, 1.88]
Monocot	12	0.0000	1.36	[1.23, 1.49]
Medium				
Hydroponic	1	0.0632	1.27	[0.99, 1.64]
Liquid	8	0.0082	1.16	[1.04, 1.30]
Soil	7	0.0000	1.68	[1.52, 1.85]
Generation				
T1	7	0.0000	1.28	[1.14, 1.44]
T2	3	0.0000	1.70	[1.48, 1.96]
T3	5	0.0000	1.31	[1.16, 1.47]
T4	1	0.0000	1.66	[1.33, 2.08]
Treatment				
Mild	6	0.0000	1.39	[1.21, 1.59]
Moderate	3	0.0000	1.57	[1.39, 1.77]
Severe	6	0.0000	1.32	[1.18, 1.47]

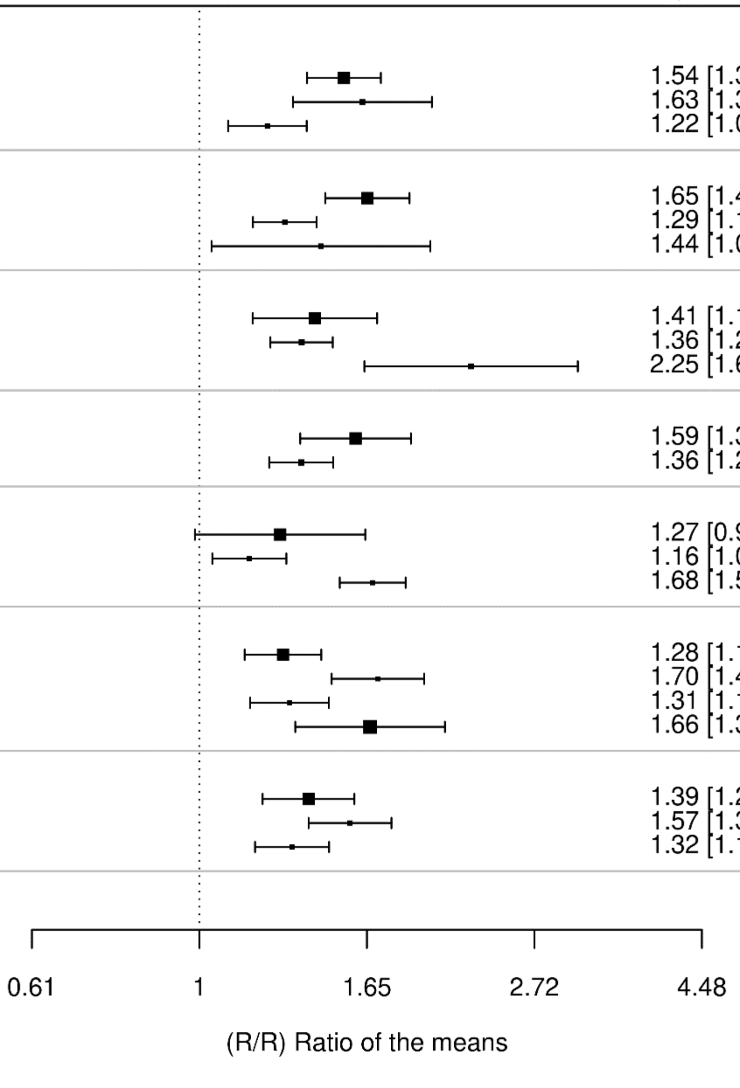

Figure S3. Seed number moderators

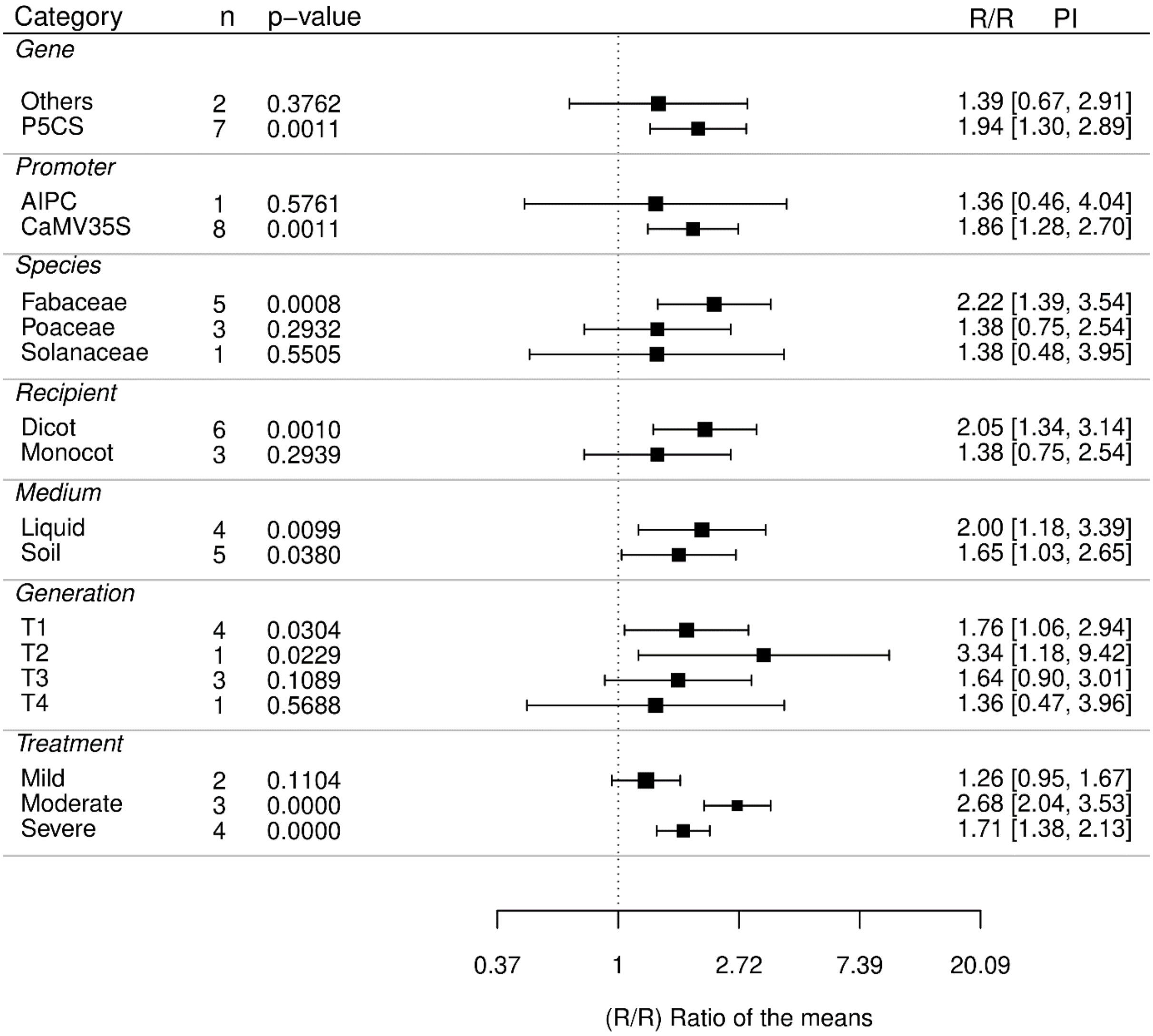

Fig. S4. Seed weight moderators

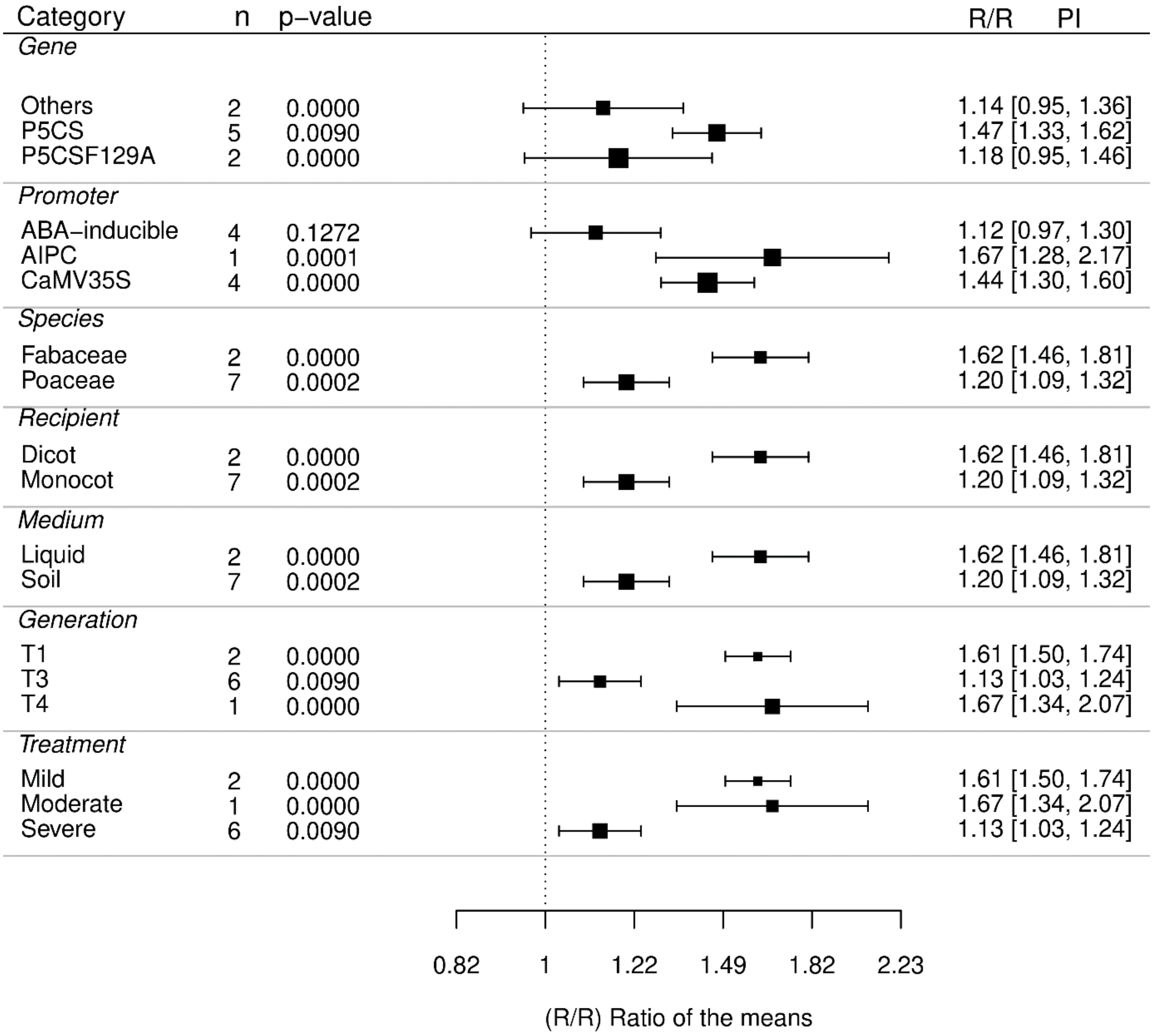

Figure S5. Chlorophyll moderators

Category	n	p−value	R/R	PI
Gene				
Others	3	0.0000	1.89	[1.62, 2.20]
P5CS	18	0.0000	1.31	[1.21, 1.41]
P5CSF129A	5	0.0032	1.24	[1.08, 1.44]
Promoter				
CaMV35S	22	0.0000	1.36	[1.26, 1.46]
Ubiquitin	4	0.0000	1.48	[1.27, 1.72]
Species				
Fabaceae	6	0.0000	1.29	[1.14, 1.45]
Others	3	0.0000	1.47	[1.23, 1.75]
Poaceae	11	0.0000	1.32	[1.19, 1.46]
Solanaceae	6	0.0000	1.57	[1.36, 1.80]
Recipient				
Dicot	15	0.0000	1.42	[1.30, 1.54]
Monocot	11	0.0000	1.32	[1.19, 1.46]
Medium				
Liquid	4	0.0015	1.33	[1.12, 1.58]
Soil	15	0.0000	1.35	[1.23, 1.48]
Solid	7	0.0000	1.53	[1.31, 1.78]
Generation				
T1	20	0.0000	1.45	[1.33, 1.58]
T2	1	0.2853	1.20	[0.86, 1.66]
T3	1	0.3884	1.15	[0.83, 1.59]
T4	4	0.0388	1.24	[1.01, 1.53]
Treatment				
Mild	6	0.0260	1.14	[1.02, 1.27]
Moderate	7	0.0000	1.39	[1.24, 1.57]
Severe	13	0.0000	1.49	[1.37, 1.62]

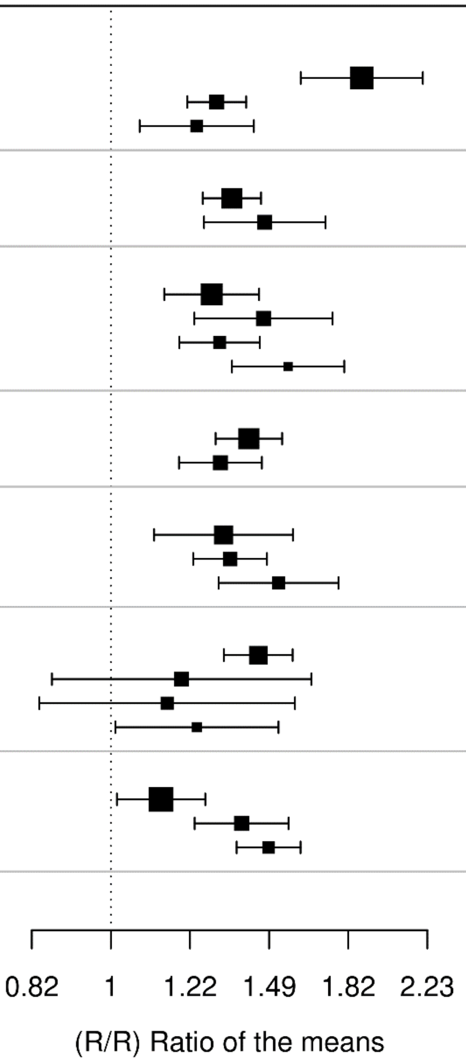

Figure S6. Root length moderators

Moderator	n	p−value	R/R PI
Gene			
OAT	6	0.0000	1.74 [1.53, 1.98]
P5CR	4	0.0000	1.57 [1.29, 1.92]
P5CS	4	0.0000	1.51 [1.26, 1.81]
Promoter			
35S x 2	4	0.0000	1.68 [1.45, 1.95]
CaMV35S	7	0.0000	1.70 [1.45, 1.99]
Other	3	0.0000	1.50 [1.24, 1.81]
Species			
Brassicaceae	3	0.0007	1.45 [1.17, 1.79]
Others	8	0.0000	2.56 [1.87, 3.51]
Poaceae	3	0.0000	1.61 [1.44, 1.80]
Recipient			
Dicot	4	0.0000	1.58 [1.29, 1.95]
Monocot	10	0.0000	1.66 [1.49, 1.85]
Medium			
Soil	5	0.0000	1.59 [1.32, 1.91]
Solid	9	0.0000	1.67 [1.49, 1.86]
Generation			
T1	3	0.0000	1.55 [1.34, 1.80]
T2	5	0.0000	1.87 [1.65, 2.12]
T3 + T4	6	0.0000	1.49 [1.35, 1.64]
Treatment			
Mild	6	0.0000	1.60 [1.43, 1.79]
Moderate	4	0.0000	1.37 [1.23, 1.53]
Severe	4	0.0000	1.80 [1.63, 1.99]

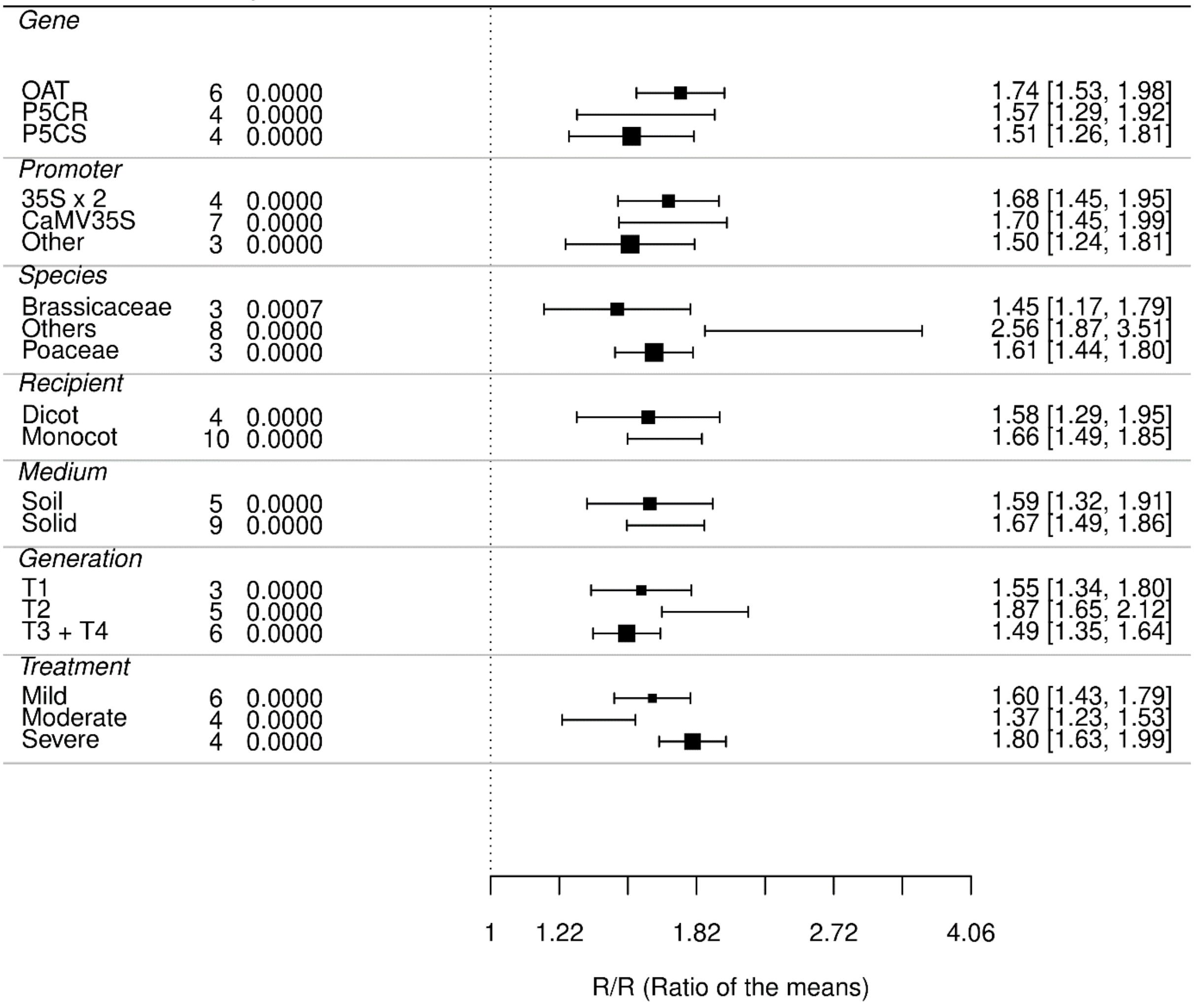

Figure S7. Plant weight moderators

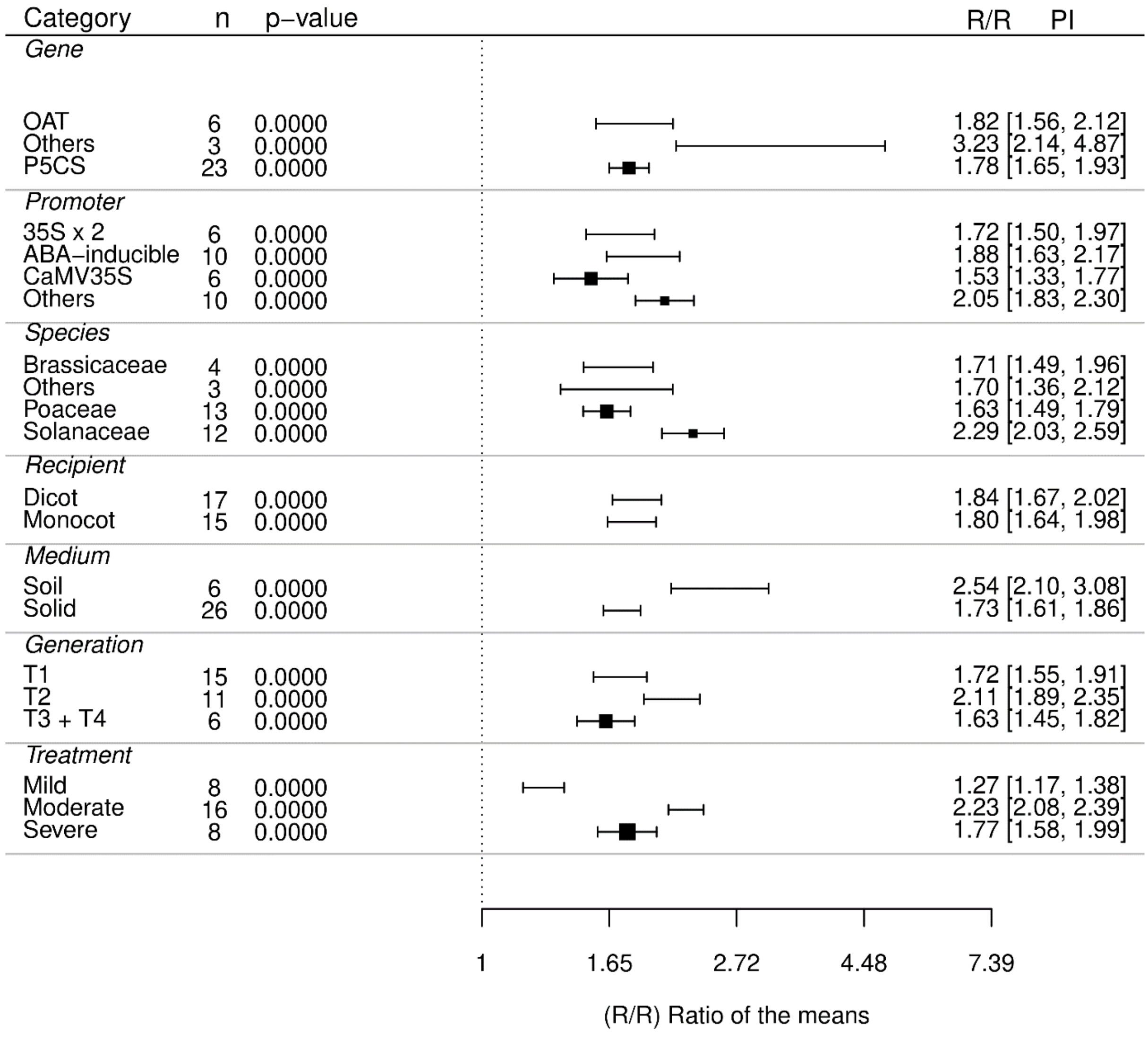

Figure S8. POD moderators

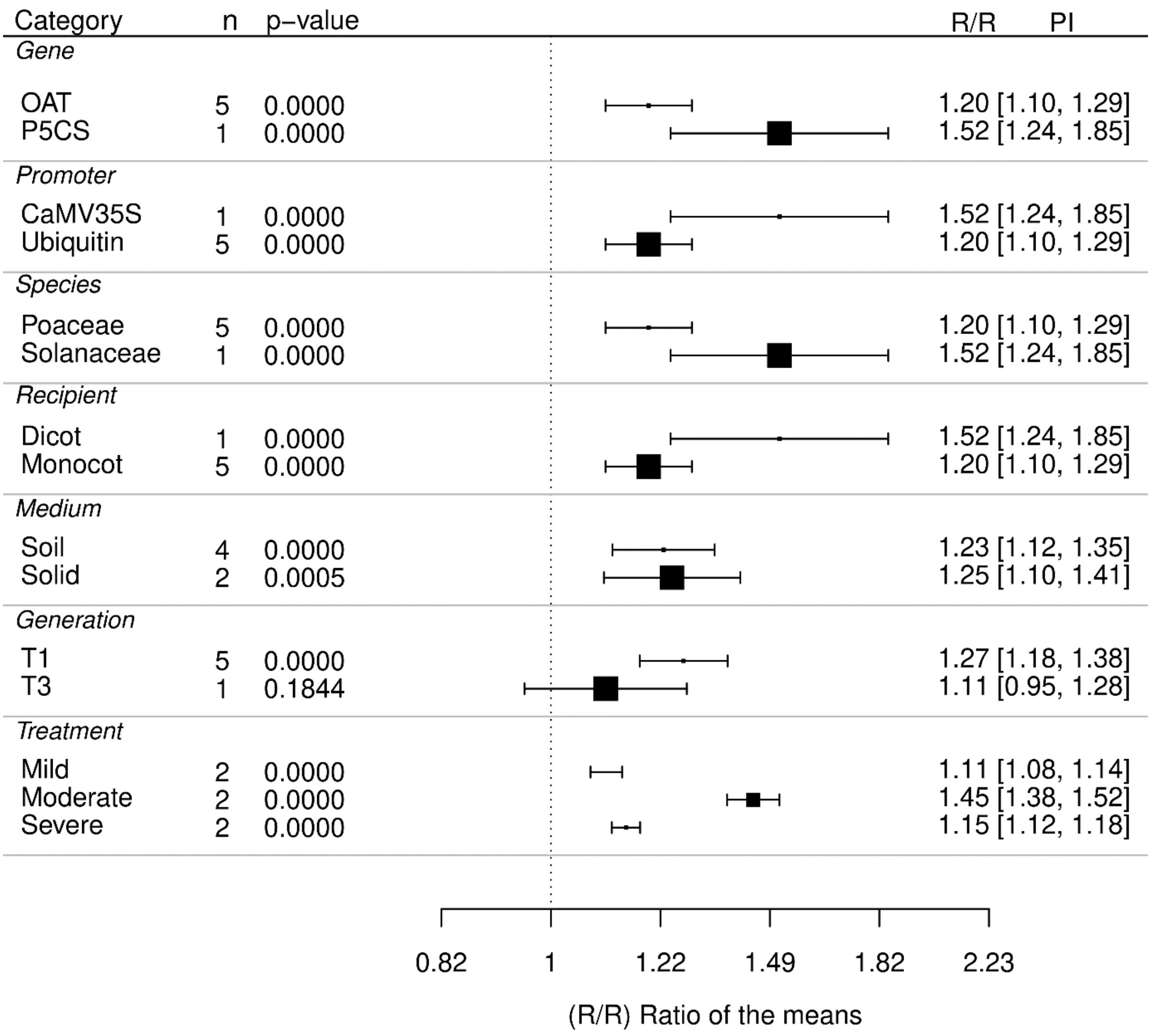

Figure S9. SOD moderators

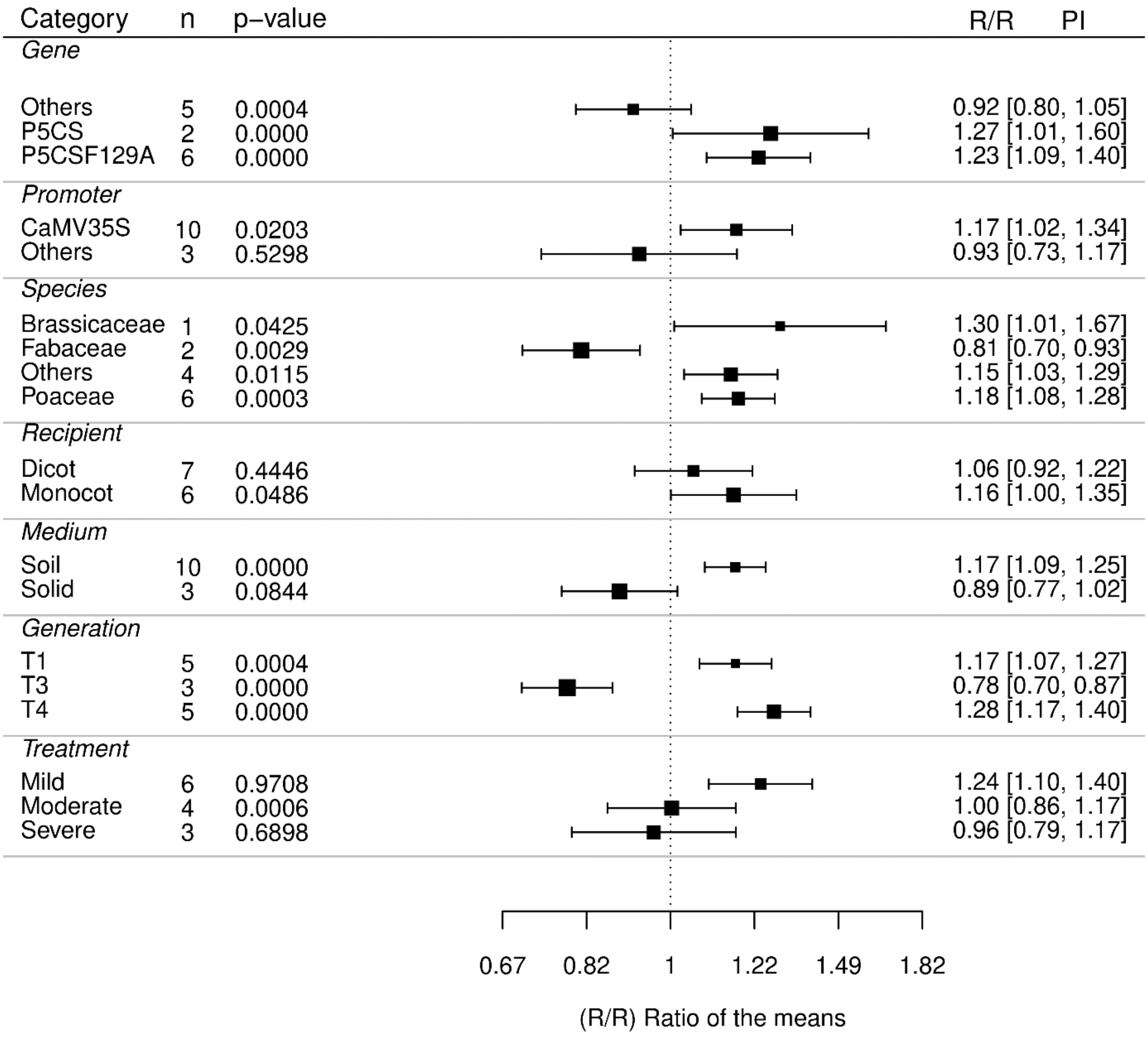

Figure S10. MDA moderators

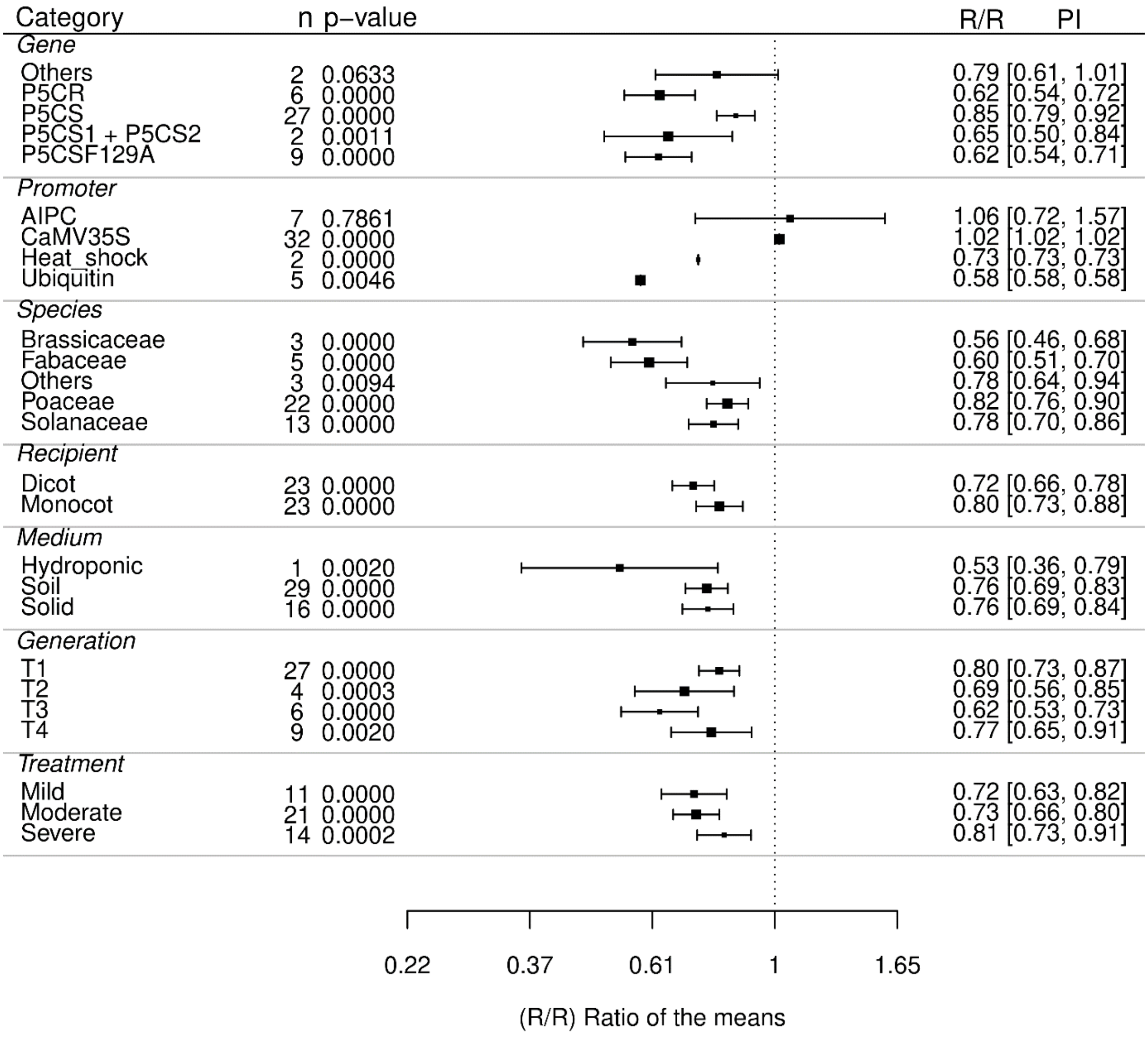

Figure S11. CAT moderators

Category	n	p–value	R/R PI
Gene			
P5CS	19	0.0044	1.37 [1.10, 1.69]
P5CSF129A	6	0.0046	1.70 [1.18, 2.45]
Species			
Poaceae	4	0.0000	2.63 [2.06, 3.36]
Rutaceae	2	0.0488	0.71 [0.51, 1.00]
Solanaceae	19	0.0000	1.32 [1.17, 1.49]
Recipient			
Dicot	21	0.0027	1.25 [1.08, 1.45]
Monocot	4	0.0000	2.63 [1.92, 3.61]
Medium			
Soil	6	0.0046	1.70 [1.18, 2.45]
Solid	19	0.0044	1.37 [1.10, 1.69]
Generation			
T1	21	0.0027	1.25 [1.08, 1.45]
T4	4	0.0000	2.63 [1.92, 3.61]
Treatment			
Mild	5	0.0000	2.34 [1.78, 3.08]
Moderate	5	0.3492	1.14 [0.87, 1.51]
Severe	15	0.0027	1.28 [1.09, 1.51]

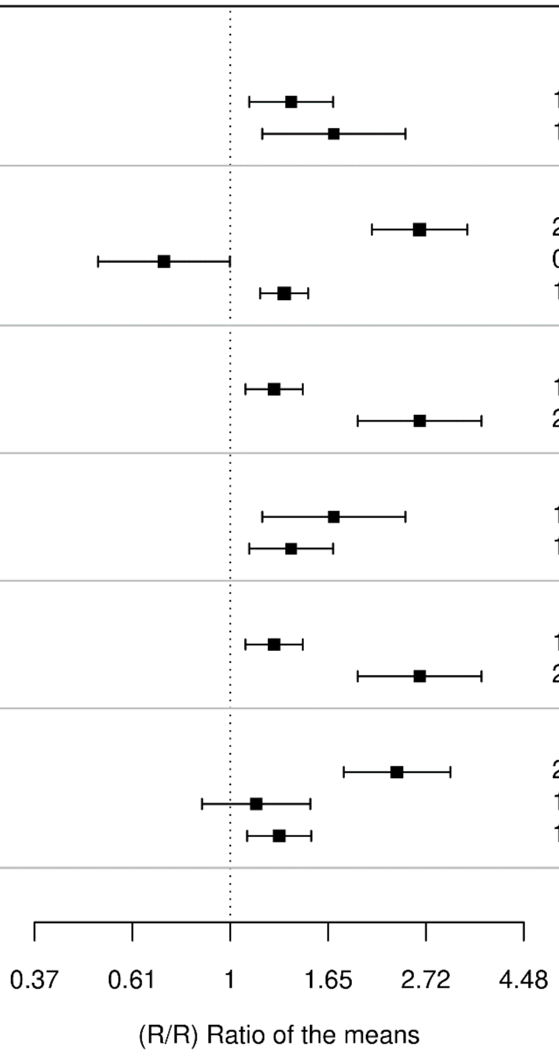

Figure S12. APX moderators

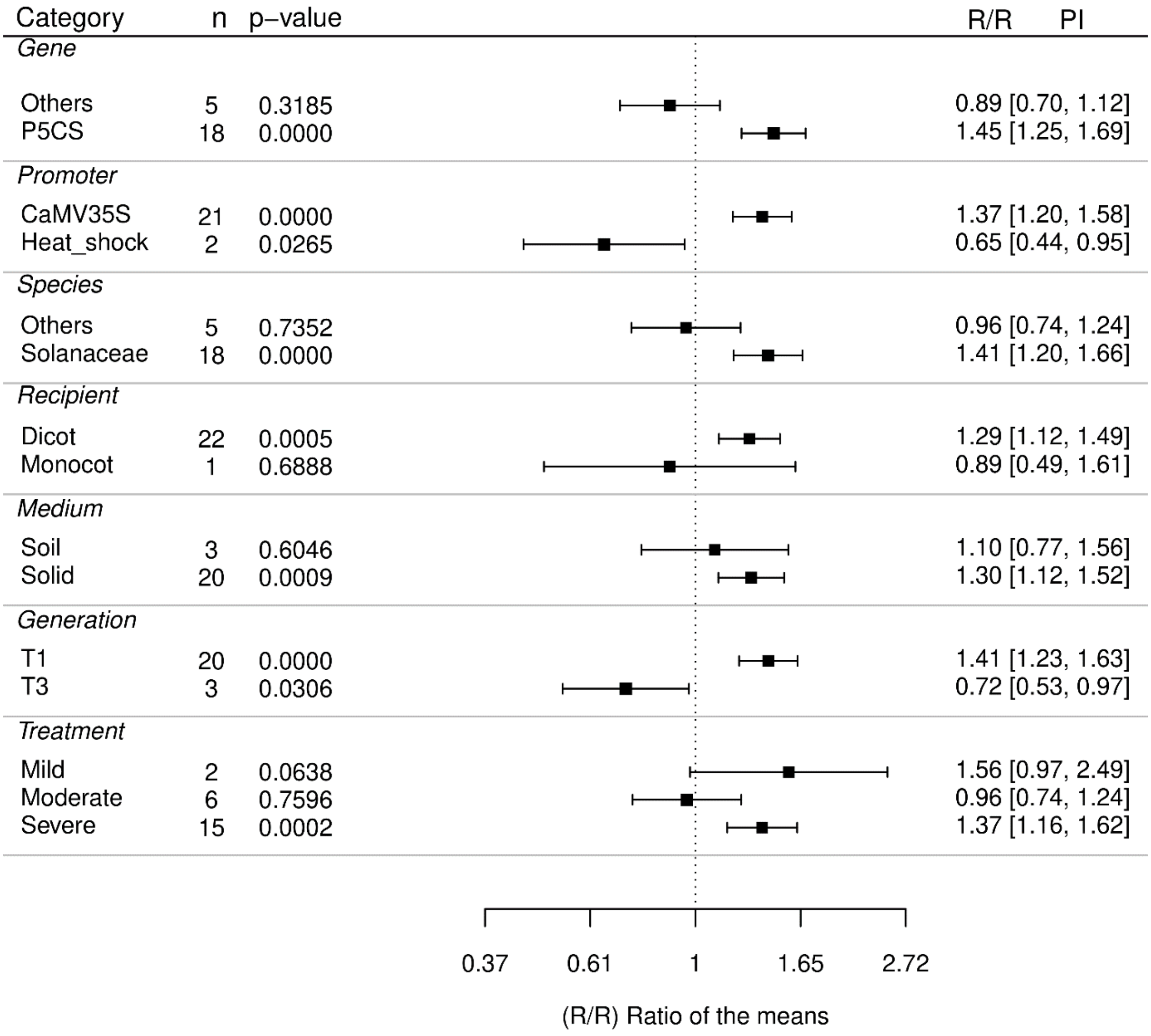

Figure S13. RWC moderators

Category	n	p–value	R/R	PI
Gene				
Others	3	0.0000	1.22	[1.18, 1.27]
P5CS	14	0.0001	1.03	[1.02, 1.05]
Promoter				
AIPC	4	0.0045	1.06	[1.02, 1.10]
CaMV35S	5	0.0017	1.05	[1.02, 1.08]
Heat_shock	2	0.0000	1.22	[1.16, 1.28]
Ubiquitin	6	0.0258	1.03	[1.00, 1.06]
Species				
Others	2	0.0000	1.22	[1.16, 1.29]
Poaceae	15	0.0000	1.04	[1.03, 1.06]
Recipient				
Dicot	3	0.0000	1.22	[1.18, 1.27]
Monocot	14	0.0001	1.03	[1.02, 1.05]
Medium				
Soil	15	0.0000	1.04	[1.03, 1.06]
Solid	2	0.0000	1.22	[1.16, 1.28]
Generation				
T1	10	0.0027	1.04	[1.01, 1.06]
T2 + T3	3	0.0000	1.16	[1.11, 1.20]
T4	4	0.0053	1.06	[1.02, 1.10]
Treatment				
Mild	2	0.3988	0.96	[0.88, 1.05]
Moderate	7	0.0000	1.07	[1.04, 1.10]
Severe	8	0.0000	1.08	[1.05, 1.12]

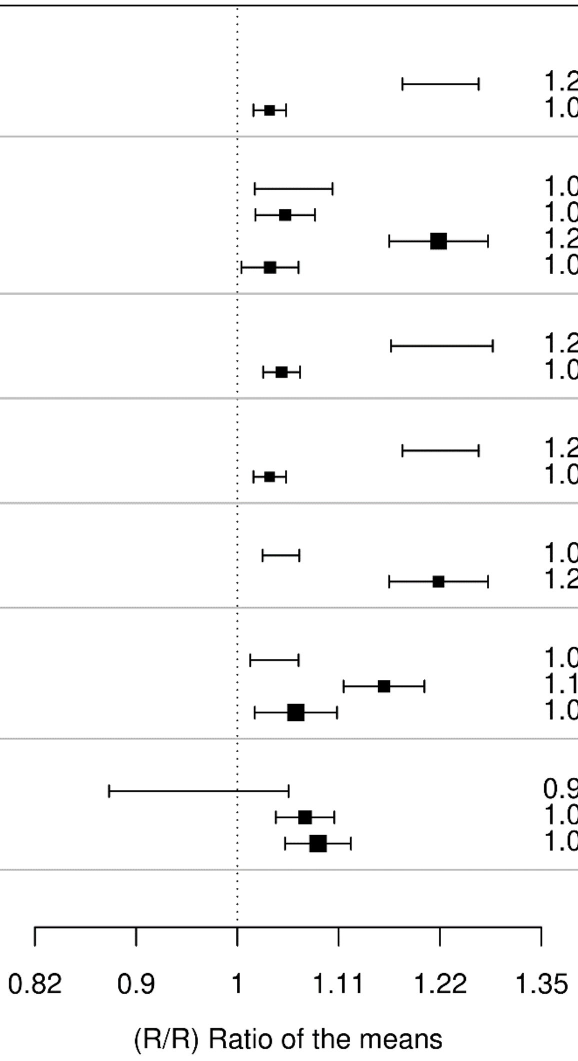

Figure S14. Stomatal aperture moderators

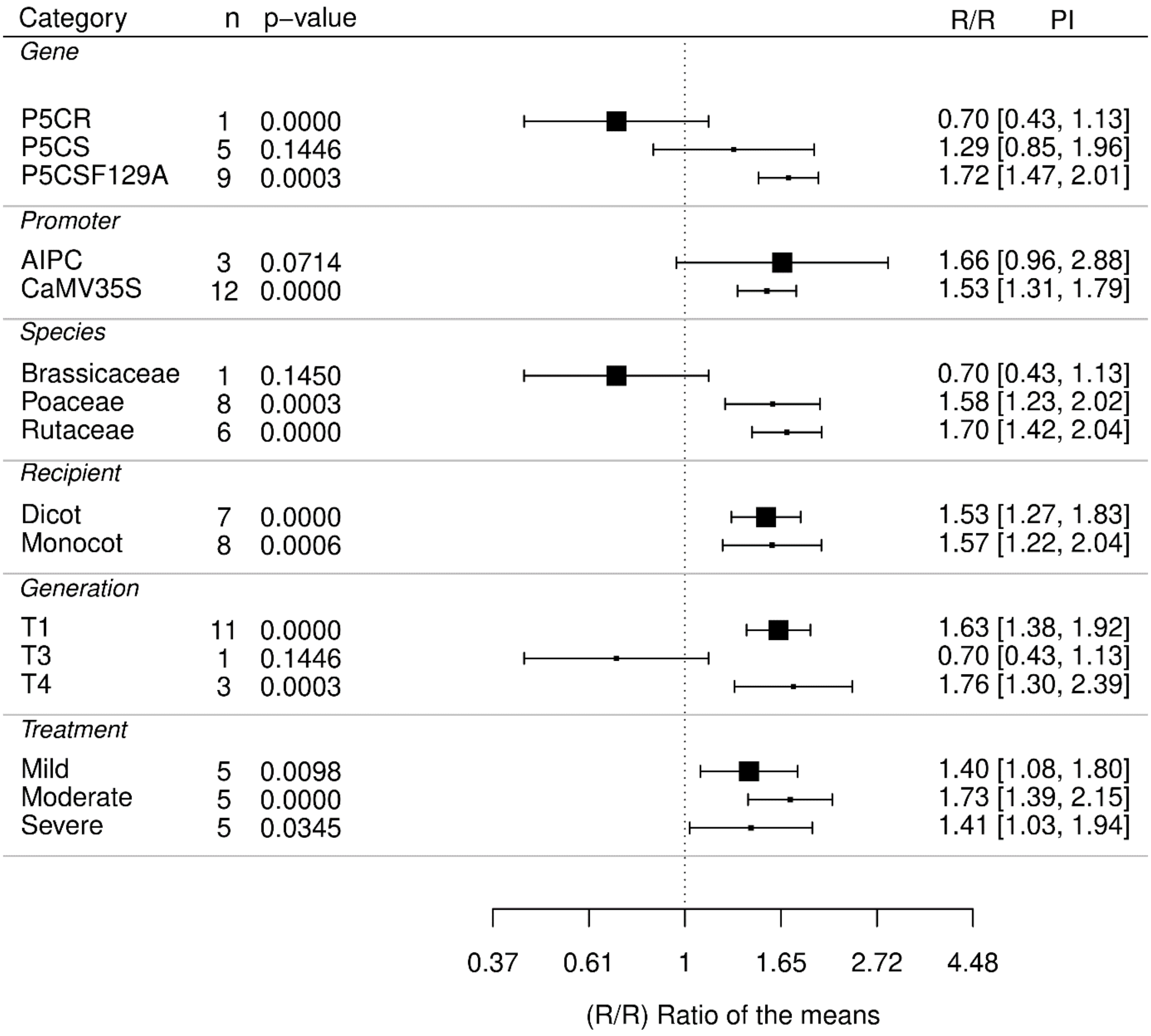

Figure S15. REC moderators

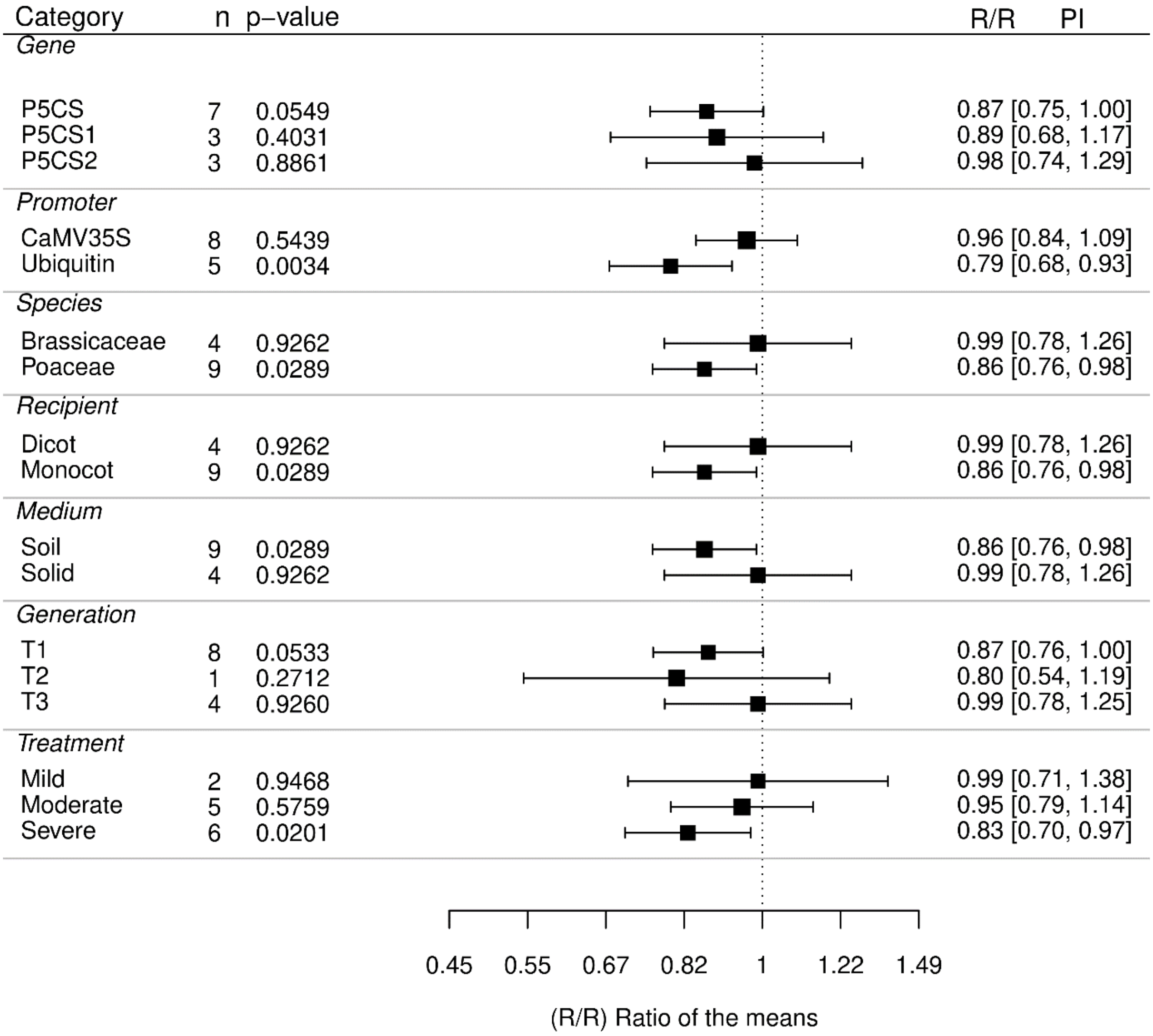

Figure S16. SUR moderators

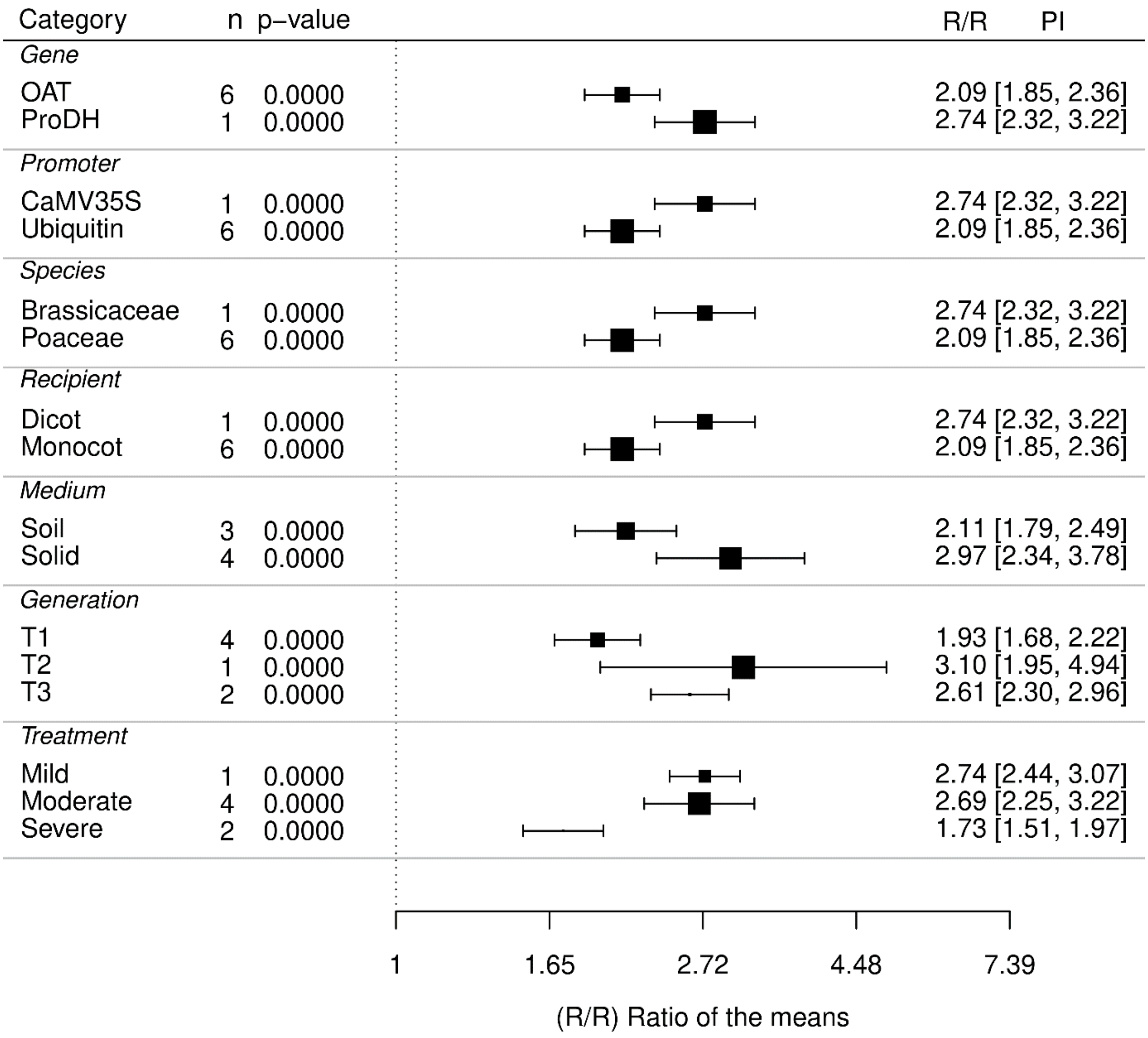

Moderation analysis under non-stress conditions

Figure S17. Proline moderators

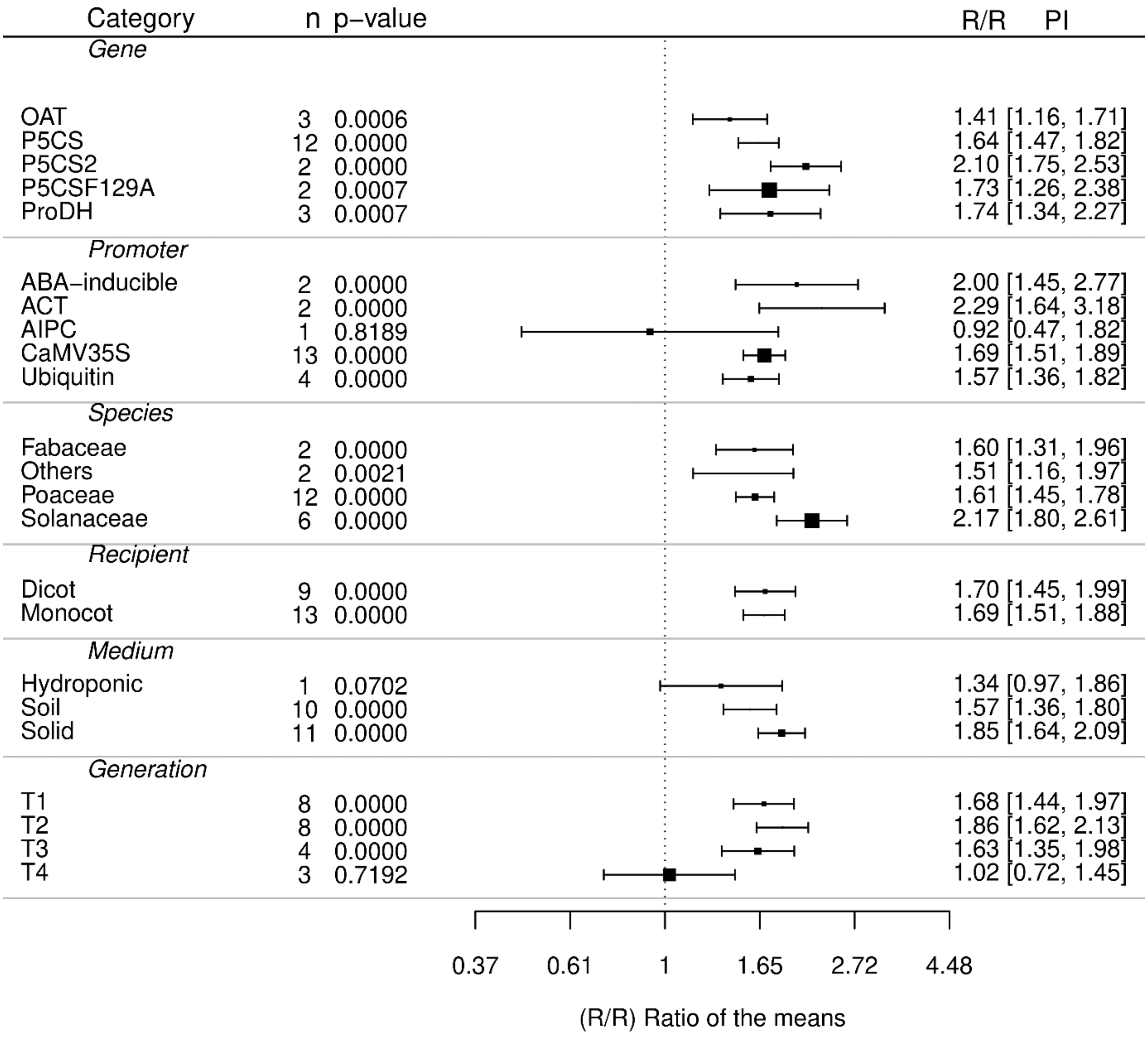

Figure S18. Plant height moderators

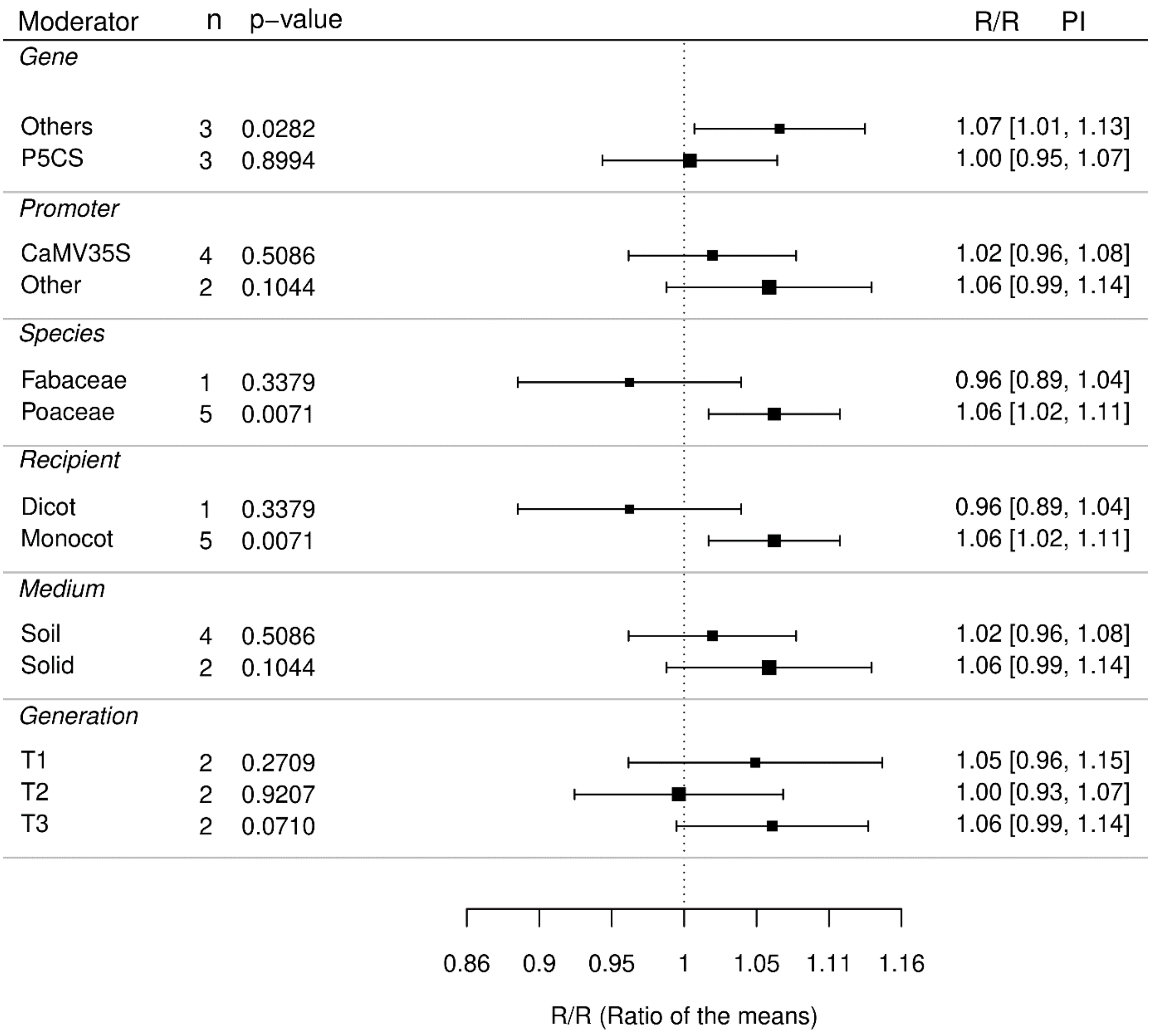

Figure S19. Seed number moderators

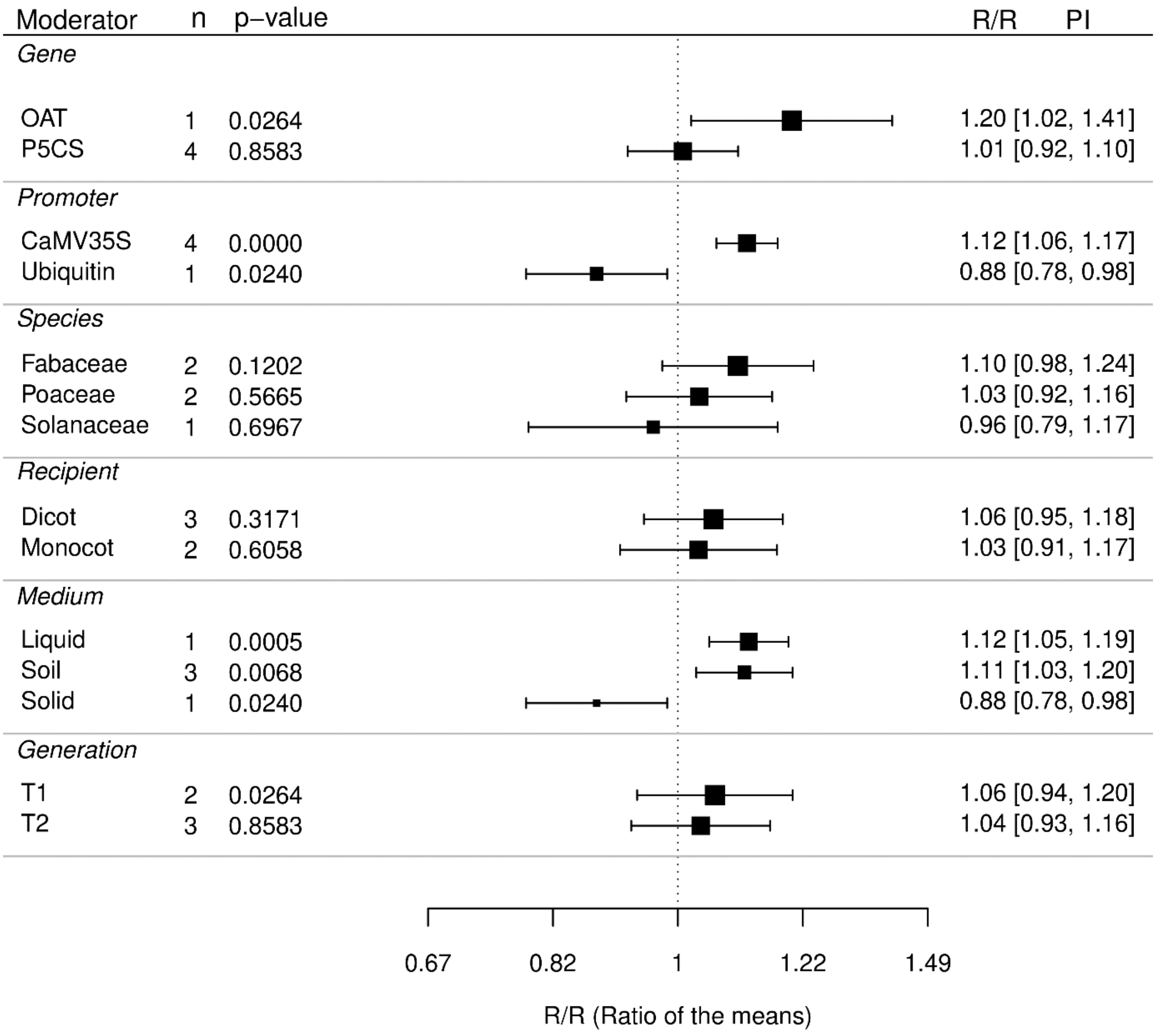

Figure S20. Seed weight moderators

Moderator	n	p–value	R/R PI
Gene			
OAT	1	0.6849	1.04 [0.85, 1.28]
P5CS	3	0.1880	1.07 [0.97, 1.18]
P5CSF129A	2	0.7315	0.97 [0.80, 1.17]
Promoter			
ABA–inducible	4	0.4368	0.95 [0.83, 1.08]
CaMV35S	2	0.0027	1.11 [1.04, 1.19]
Species			
Fabaceae	1	0.0025	1.12 [1.04, 1.21]
Poaceae	5	0.7068	0.98 [0.88, 1.09]
Recipient			
Dicot	1	0.0025	1.12 [1.04, 1.21]
Monocot	5	0.7068	0.98 [0.88, 1.09]
Medium			
Liquid	1	0.0025	1.12 [1.04, 1.21]
Soil	5	0.7068	0.98 [0.88, 1.09]
Generation			
T1	1	0.0003	1.12 [1.05, 1.20]
T2	1	0.6459	1.04 [0.87, 1.25]
T3	4	0.4419	0.95 [0.84, 1.08]

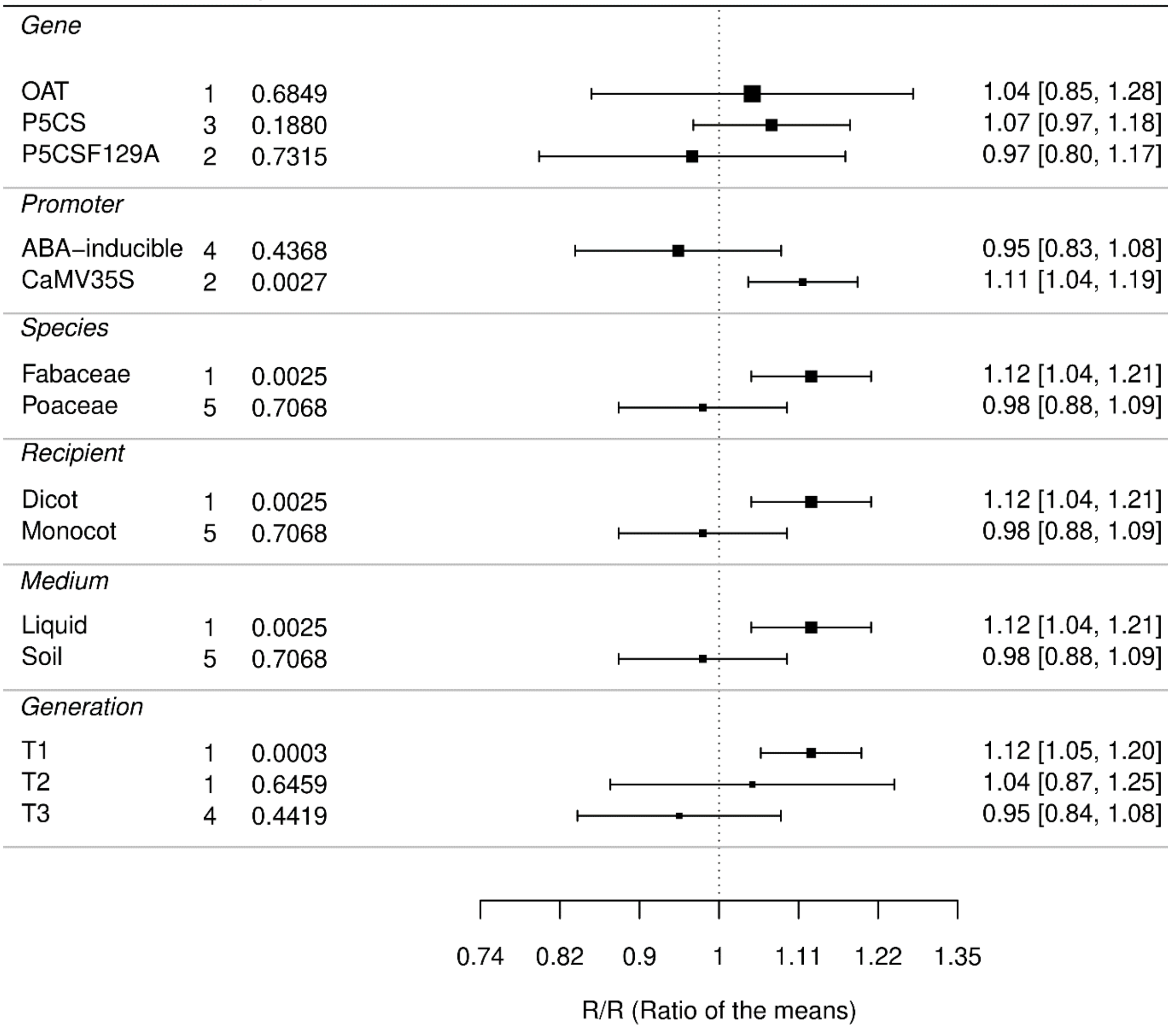

Figure S21. Chlorophyll moderators

Moderator	n	p–value	R/R	PI
Gene				
Others	4	0.3965	1.02	[0.98, 1.06]
P5CS	8	0.1223	1.04	[0.99, 1.09]
Promoter				
CaMV35S	10	0.0009	1.04	[1.02, 1.07]
Ubiquitin	2	0.1597	0.98	[0.95, 1.01]
Species				
Fabaceae	2	0.4329	1.02	[0.97, 1.08]
Others	2	0.2956	1.03	[0.98, 1.08]
Poaceae	5	0.2445	0.98	[0.96, 1.01]
Solanaceae	3	0.0009	1.06	[1.02, 1.10]
Recipient				
Dicot	7	0.0026	1.04	[1.02, 1.08]
Monocot	5	0.3476	0.98	[0.95, 1.02]
Medium				
Liquid	1	0.0226	1.22	[1.03, 1.45]
Soil	8	0.4753	1.01	[0.99, 1.03]
Solid	3	0.0026	1.17	[1.06, 1.30]
Generation				
T1	10	0.1037	1.03	[0.99, 1.06]
T2	1	1.0000	1.00	[0.92, 1.09]
T4	1	0.4316	1.06	[0.92, 1.21]

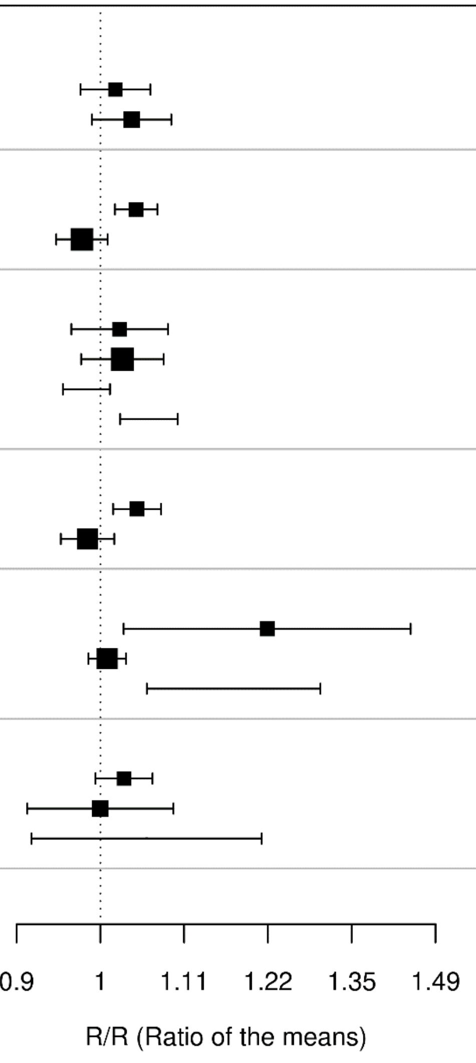

Figure S22. Root length moderators

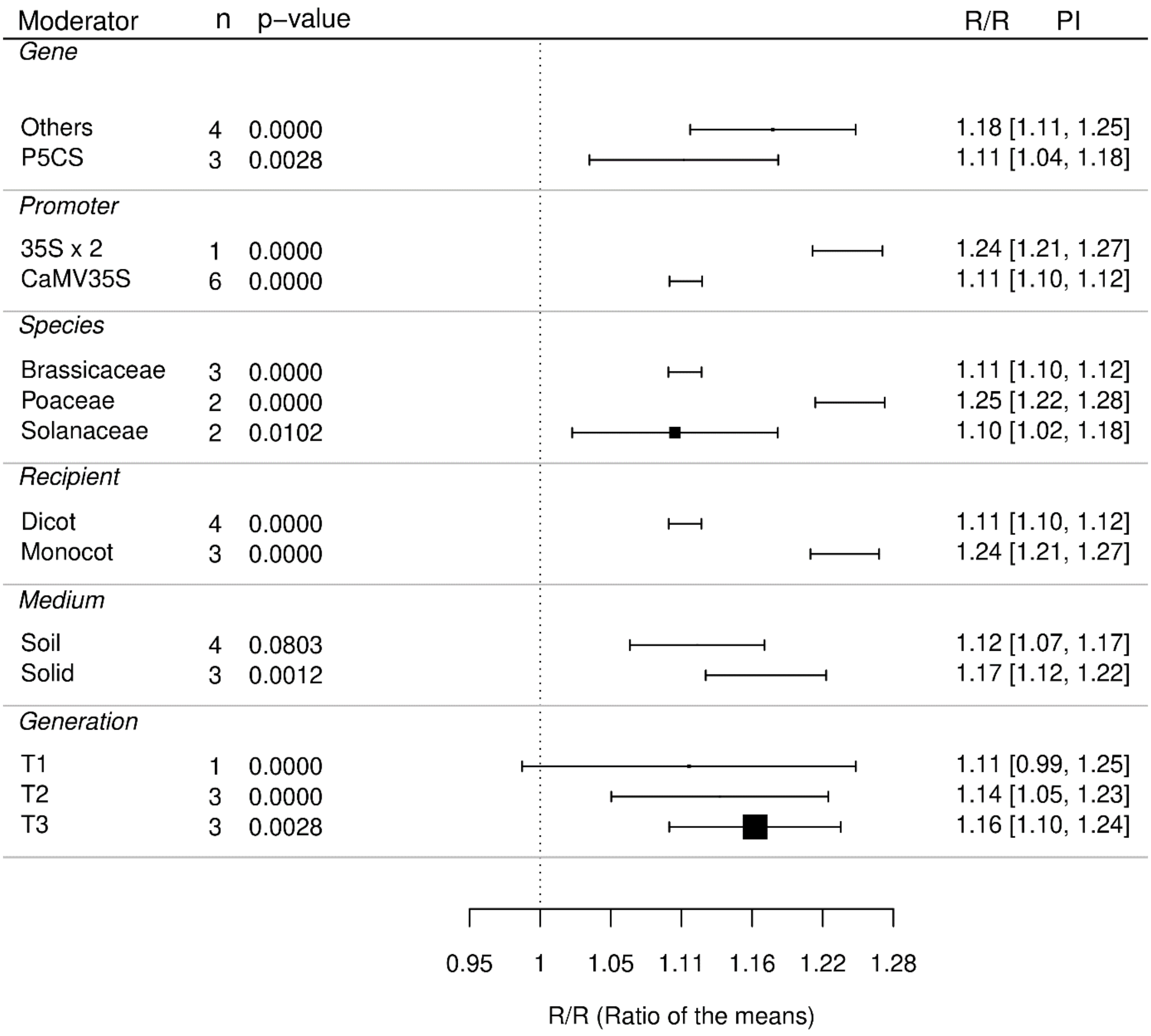

Figure S23. Plant weight moderators

Moderator	n	p−value	R/R PI
Gene			
OAT	2	0.0000	1.11 [1.05, 1.16]
Others	3	0.0000	0.97 [0.96, 0.98]
P5CS	4	0.2223	0.99 [0.98, 1.01]
Promoter			
35S x 2	1	0.0003	1.10 [1.05, 1.16]
CaMV35S	8	0.0447	0.98 [0.97, 1.00]
Species			
Brassicaceae	2	0.0000	0.98 [0.97, 0.99]
Others	2	0.0000	1.11 [1.05, 1.16]
Solanaceae	5	0.1750	1.05 [0.98, 1.14]
Recipient			
Dicot	7	0.0435	0.98 [0.97, 1.00]
Monocot	2	0.0003	1.10 [1.05, 1.16]
Medium			
Soil	1	0.8830	0.99 [0.87, 1.13]
Solid	8	0.3536	1.01 [0.99, 1.04]
Generation			
T1	5	0.0715	1.07 [0.99, 1.15]
T2	1	0.8475	1.04 [0.69, 1.58]
T3	3	0.9330	1.00 [0.97, 1.03]

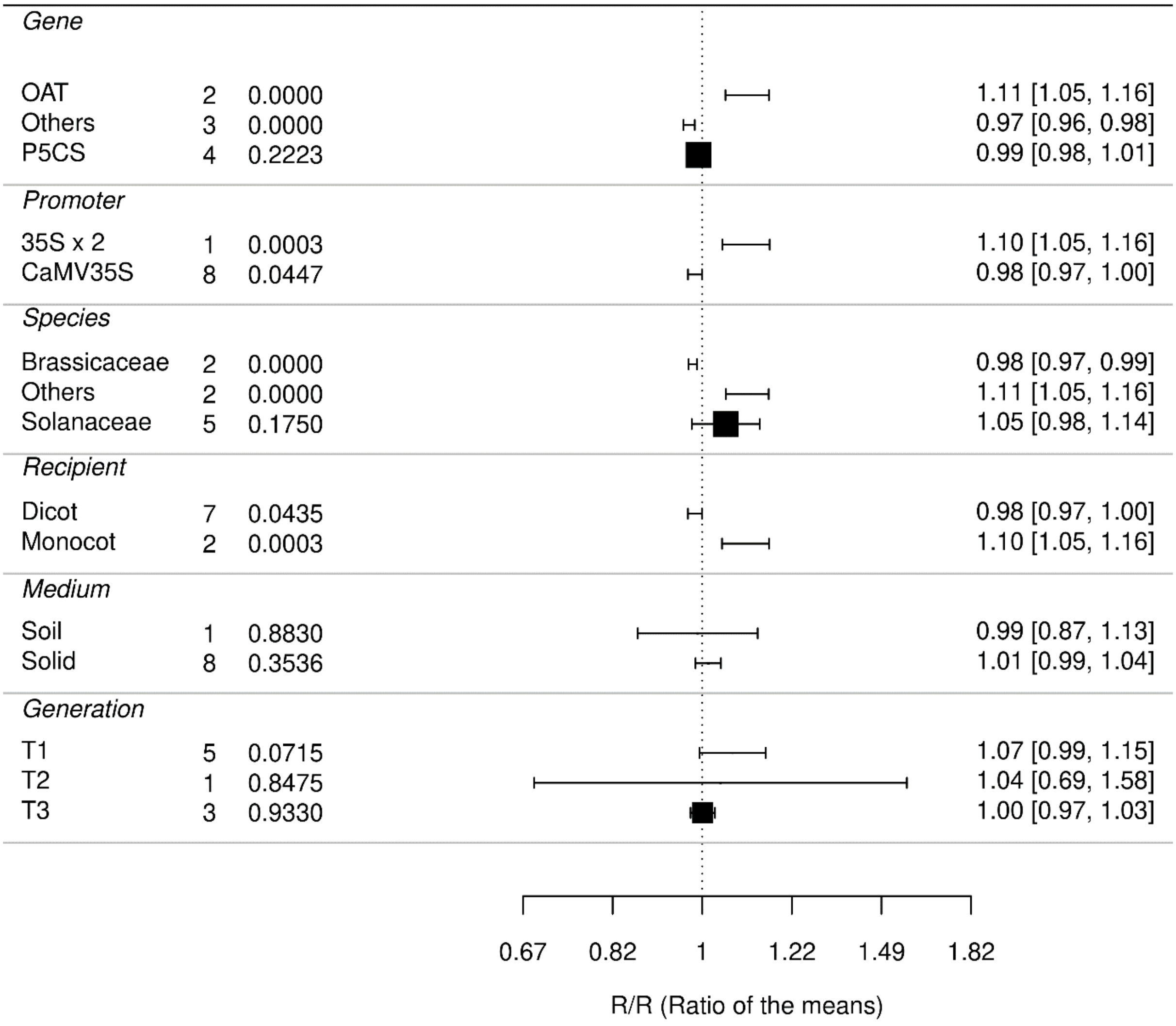

Figure S24. SOD moderators

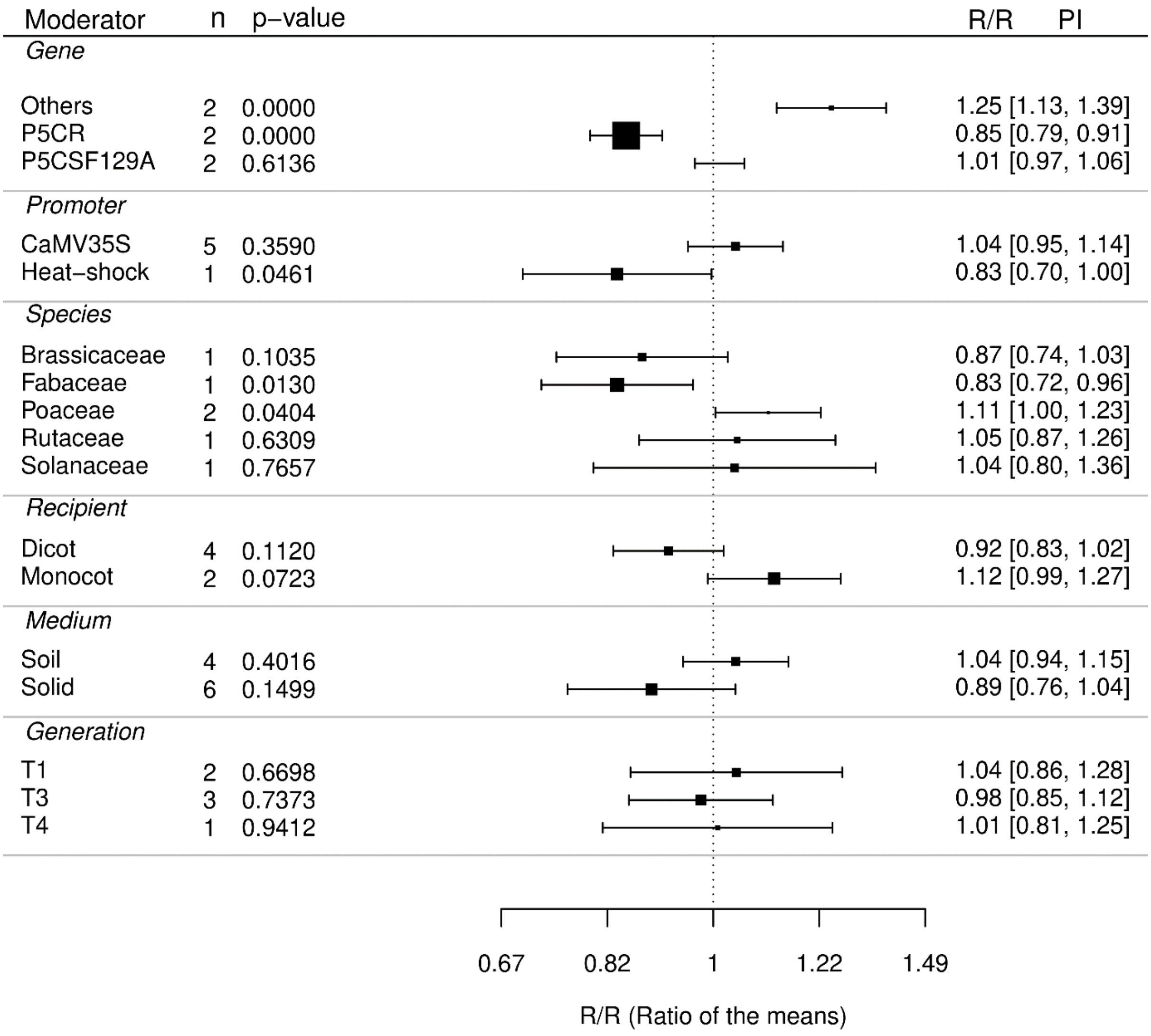

Figure S25. MDA moderators

Moderator	n	p–value	R/R PI
Gene			
Others	2	0.4777	0.98 [0.94, 1.03]
P5CR	3	0.0000	0.88 [0.84, 0.93]
P5CS	11	0.0000	1.13 [1.09, 1.17]
P5CSF129A	3	0.6497	0.95 [0.76, 1.18]
Promoter			
AIPC	2	0.0000	1.18 [1.15, 1.22]
CaMV35S	15	0.3068	0.98 [0.95, 1.02]
Others	2	0.0000	0.88 [0.85, 0.92]
Species			
Brassicaceae	2	0.3984	0.95 [0.84, 1.07]
Fabaceae	3	0.0000	0.90 [0.86, 0.95]
Poaceae	6	0.0000	1.16 [1.12, 1.20]
Rutaceae	1	0.8882	1.03 [0.72, 1.46]
Solanaceae	7	0.2056	0.97 [0.93, 1.02]
Recipient			
Dicot	12	0.0000	0.92 [0.88, 0.95]
Monocot	7	0.0000	1.12 [1.08, 1.16]
Medium			
Hydroponic	1	0.1092	1.14 [0.97, 1.33]
Soil	12	0.4450	1.02 [0.97, 1.07]
Solid	6	0.0425	0.94 [0.88, 1.00]
Generation			
T1	11	0.1957	1.03 [0.98, 1.09]
T2	2	0.5899	1.03 [0.94, 1.12]
T3	4	0.0420	0.93 [0.87, 1.00]
T4	2	0.3799	0.92 [0.77, 1.10]

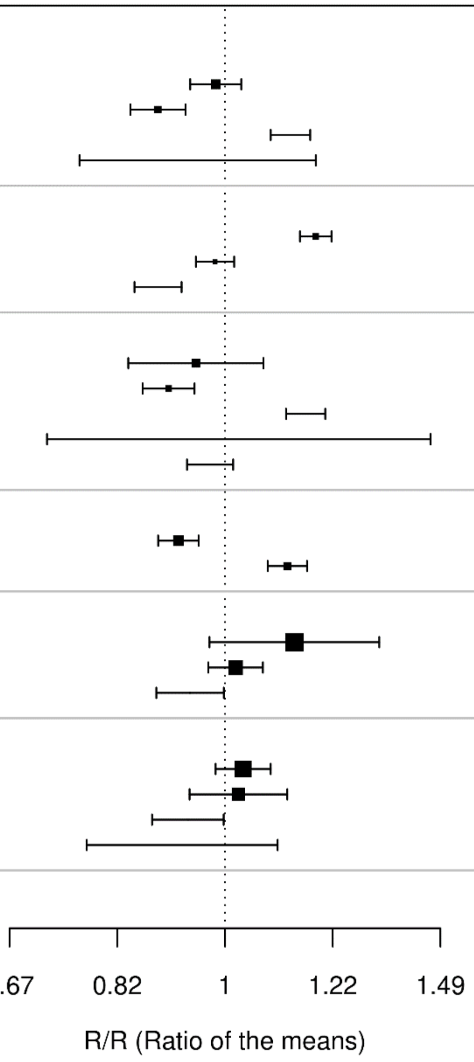

Figure S26. CAT moderators

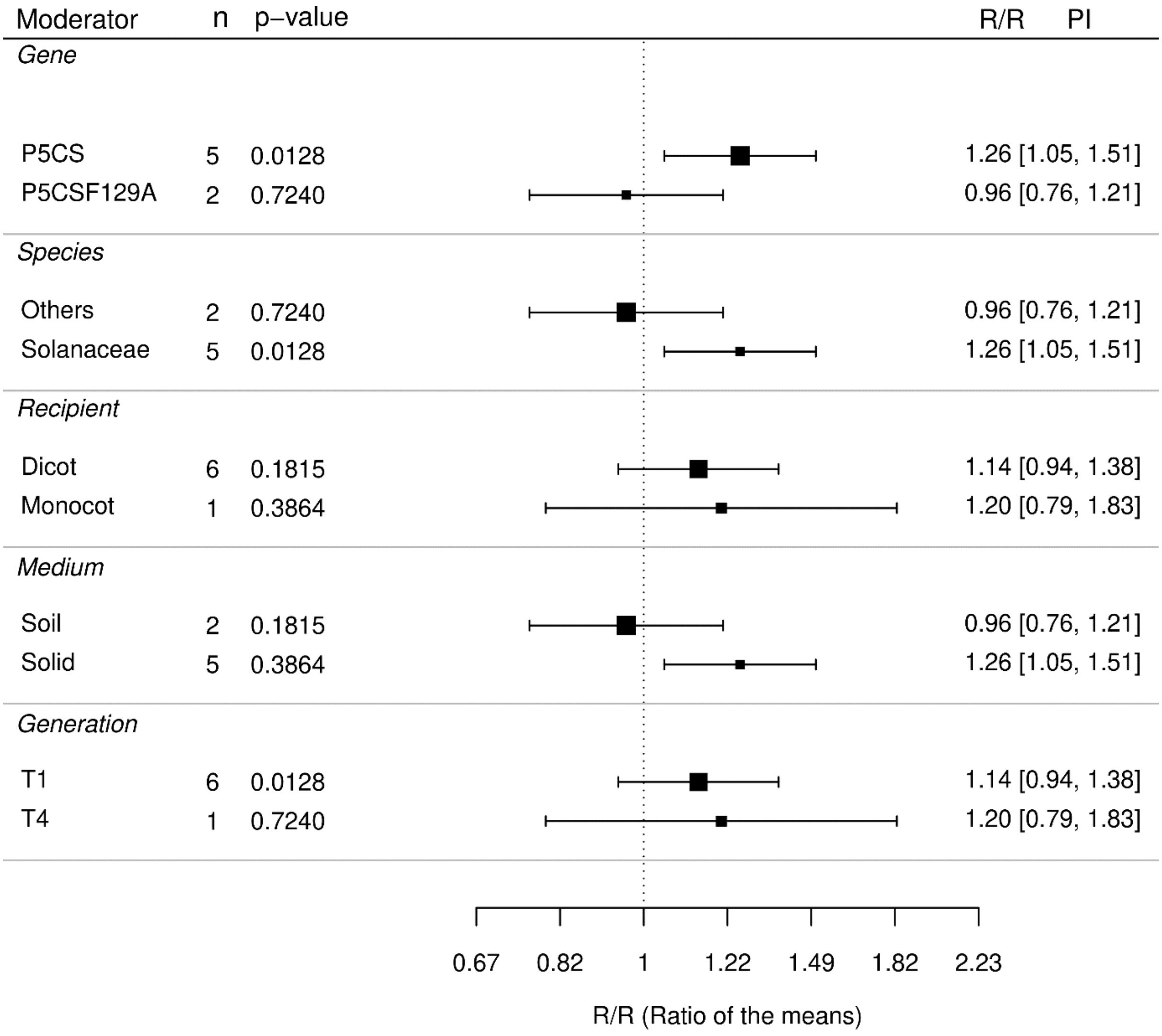

Figure S27. APX moderators

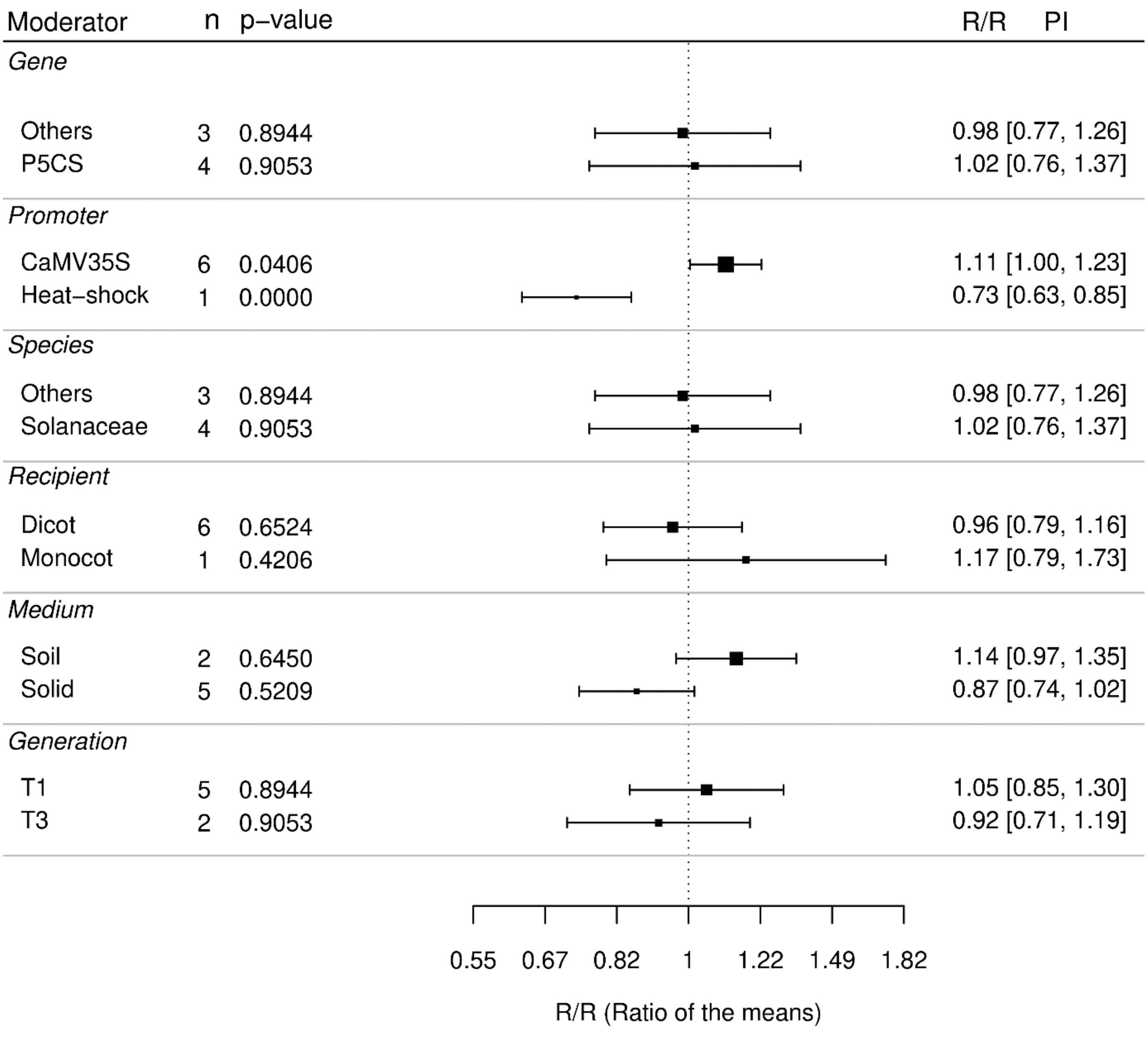

Figure S28. Sto moderators

Moderator	n	p–value	R/R PI
Gene			
P5CS	3	0.6203	0.99 [0.94, 1.04]
P5CSF129A	2	0.0209	1.17 [1.02, 1.35]
Promoter			
AIPC	1	0.0704	1.51 [0.97, 2.37]
CaMV35S	4	0.5577	1.02 [0.96, 1.09]
Species			
Poaceae	4	0.5863	1.02 [0.95, 1.10]
Rutaceae	1	0.1420	1.21 [0.94, 1.56]
Recipient			
Dicot	1	0.1420	1.21 [0.94, 1.56]
Monocot	4	0.5863	1.02 [0.95, 1.10]
Generation			
T1	4	0.7717	1.01 [0.94, 1.08]
T4	1	0.1099	1.16 [0.97, 1.39]

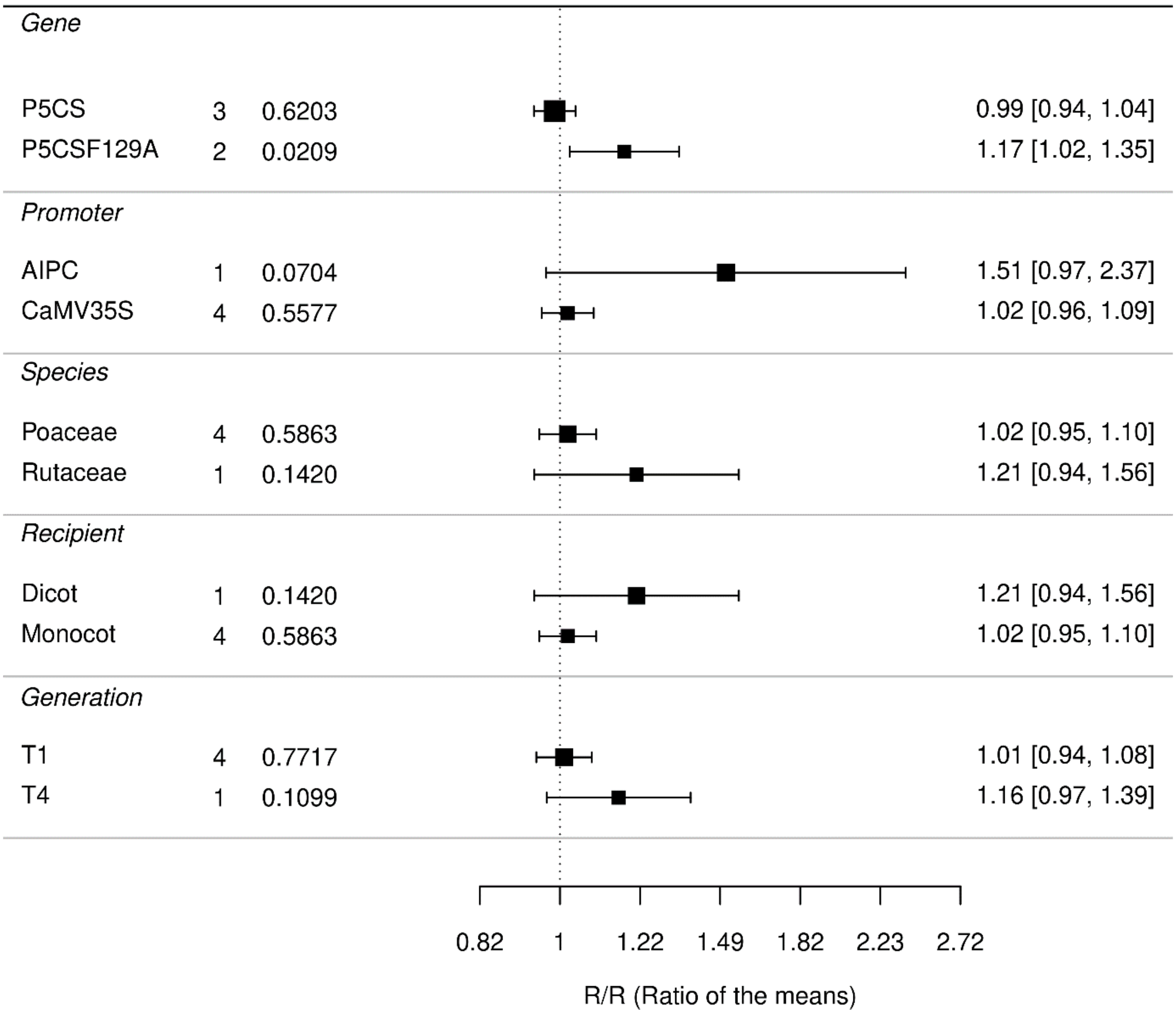

Supplement: Supplementary file 1 [file plants-13-01913-s001.zip › Figures_S1-S28.pdf]
