# Supplementary material for: Proline Metabolism Genes in Transgenic Plants: Meta-Analysis under Drought and Salt Stress
Source: Plants (Basel). 2024 Jul 11;13(14):1913. doi: 10.3390/plants13141913 (PMC11280441; doi:10.3390/plants13141913)
Supplement: Supplementary file 1 [file plants-13-01913-s001.zip › Tables_S1-S28.pdf]

STRESS

Table S1. Moderation effects on **proline** under drought and salt stress

Moderator	τ^2	R^2	QM p-value	Permutest
**Donor gene**	0.3054	21.82%	**0.0159**	ns
**Promoter**	0.2761	29.31%	**< .0001**	***
**Recipient host**	0.2880	26.26%	**0.0114**	*
**Recipient group**	0.3601	7.81%	0.1579	ns
**Medium**	0.3766	3.58%	0.8343	ns
**Generation**	0.3523	9.80%	0.2374	ns
**Treatment**	0.3484	10.81%	0.9145	ns

Table S2. Moderation effects on **plant height** under drought and salt stress

Moderator	τ^2	R^2	QM p-value	Permutest
**Donor gene**	0.0169	27.96%	**0.0074**	ns
**Promoter**	0.0164	30.12%	**0.0092**	ns
**Recipient host**	0.0202	13.64%	**0.0115**	ns
**Recipient group**	0.0217	7.62%	**< .0001**	ns
**Medium**	0.0145	38.01%	**< .0001**	ns
**Generation**	0.0128	45.19%	**0.0037**	ns
**Treatment**	0.0146	37.71%	0.1175	ns

Table S3. Moderation effects on **seed number**

Moderator	τ^2	R^2	QM p-value	Permutest
**Donor gene**	0.2759	2.07%	0.4403	ns
**Promoter**	0.2813	0.15%	0.5961	ns
**Recipient host**	0.2745	2.54%	0.4211	ns
**Recipient group**	0.2754	2.24%	0.3004	ns
**Medium**	0.2798	0.70%	0.5944	ns
**Generation**	0.2699	4.19%	0.6335	ns
**Treatment**	0.0413	85.34%	**0.0008**	ns

Table S4. Moderation effects on **seed weight**

Moderator	τ^2	R^2	QM p-value	Permutest
**Donor gene**	0.0077	50.13%	**0.0196**	ns
**Promoter**	0.0079	49.32%	**0.0059**	ns
**Recipient host**	0.0053	65.61%	**0.0000**	*
**Recipient group**	0.0053	65.61%	**0.0000**	*
**Medium**	0.0053	65.61%	**0.0000**	*
**Generation**	0.0021	86.73%	**0.0000**	*
**Treatment**	0.0021	86.73%	**0.0005**	*

Table S5. Moderation effects on **chlorophyll**

Moderator	τ^2	R^2	QM p-value	Permutest
**Donor gene**	0.0128	48.75%	**< .0001**	*
**Promoter**	0.0171	31.75%	0.3387	ns
**Recipient host**	0.0147	41.21%	0.1354	ns
**Recipient group**	0.0163	34.65%	0.2948	ns
**Medium**	0.0219	12.37%	0.3534	ns
**Generation**	0.0249	0.47%	0.2433	ns
**Treatment**	0.0105	57.81%	**0.0005**	ns

Table S6. Moderation effects on **root lenght**

Moderator	τ^2	R^2	QM p-value	Permutest
**Donor gene**	0.0223	20.24%	0.4088	ns
**Promoter**	0.0226	19.24%	0.5616	ns
**Recipient host**	0.0238	15.30%	**0.0107**	ns
**Recipient group**	0.0241	14.76%	0.6863	ns
**Medium**	0.0241	13.76%	0.6746	ns
**Generation**	0.0111	60.41%	**0.0176**	ns
**Treatment**	0.0082	70.48%	**0.0011**	ns

Table S7. Moderation effects on **plant weight**

Moderator	τ^2	R^2	QM p-value	Permutest
**Donor gene**	0.0236	10.52%	**0.0206**	ns
**Promoter**	0.0218	17.36%	**0.0164**	ns
**Recipient host**	0.0183	30.58%	**0.0002**	ns
**Recipient group**	0.0231	12.47%	0.7678	ns
**Medium**	0.0235	10.88%	**0.0002**	ns
**Generation**	0.0189	28.52%	**0.0029**	ns
**Treatment**	0.0086	67.35%	**< .0001**	**

Table S8. Moderation effects on **peroxidase activity (POD)**

Moderator	τ^2	R^2	QM p-value	Permutest
**Donor gene**	0.0060	18.37%	**0.0286**	ns
**Promoter**	0.0060	18.37%	**0.0286**	ns
**Recipient host**	0.0060	18.37%	**0.0286**	ns
**Recipient group**	0.0060	18.37%	**0.0286**	ns
**Medium**	0.0063	14.61%	0.8465	ns
**Generation**	0.0055	25.76%	0.0975	ns
**Treatment**	0.000	100.00%	**< .0001**	ns

Table S9. Moderation effects on **superoxide dismutase (SOD)**

Moderator	τ^2	R^2	QM p-value	Permutest
**Donor gene**	0.0220	59.99%	**0.0031**	ns
**Promoter**	0.0421	23.35%	0.0903	ns
**Recipient host**	0.0097	82.28%	**< .0001**	ns
**Recipient group**	0.0325	40.88%	0.3615	ns
**Medium**	0.0115	79.10%	**0.0005**	ns
**Generation**	0.0073	86.64%	**< .0001**	**
**Treatment**	0.0223	59.39%	0.0327	ns

Table S10. Moderation effects on **malondialdehyde (MDA)**

Moderator	τ^2	R^2	QM p-value	Permutest
**Donor gene**	0.0288	33.79%	**< .0001**	*
**Promoter**	0.0251	42.28%	**0.0058**	ns
**Recipient host**	0.0254	41.58%	**0.0002**	*
**Recipient group**	0.0361	16.87%	0.0997	ns
**Medium**	0.0391	10.08%	0.2261	ns
**Generation**	0.0361	16.91%	**0.0461**	ns
**Treatment**	0.0355	18.40%	0.2316	ns

Table S11. Moderation effects on **catalase activity (CAT)**

Moderator	τ^2	R^2	QM p-value	Permutest
**Donor gene**	0.2011	1.64%	0.3136	ns
**Promoter**	0.0529	74.11%	**< .0001**	***
**Recipient host**	0.0953	53.39%	**< .0001**	***
**Recipient group**	0.2011	1.64%	0.3136	ns
**Medium**	0.0953	53.39%	**< .0001**	***
**Generation**	0.0825	59.65%	**0.0002**	***
**Treatment**	0.0086	67.35%	**< .0001**	**

Table S12. Moderation effects on **ascorbate peroxidase activity (APX)**

Moderator	τ^2	R^2	QM p-value	Permutest
**Donor gene**	0.0695	23.54%	**0.0006**	*
**Promoter**	0.0736	18.99%	**0.0003**	ns
**Recipient host**	0.0883	2.82%	0.2272	ns
**Recipient group**	0.0883	2.82%	0.2272	ns
**Medium**	0.0909	0.01%	0.3750	ns
**Generation**	0.0668	26.54%	**< .0001**	*
**Treatment**	0.0777	14.53%	**0.0479**	ns

Table S13. Moderation effects on **relative water content (RWC)**

Moderator	τ^2	R^2	QM p-value	Permutest
**Donor gene**	0.0004	67.41%	**< .0001**	**
**Promoter**	0.0006	49.64%	**< .0001**	ns
**Recipient host**	0.0006	47.49%	**< .0001**	*
**Recipient group**	0.0004	67.41%	**< .0001**	**
**Medium**	0.0006	48.68%	**< .0001**	*
**Generation**	0.0008	35.41%	**< .0001**	ns
**Treatment**	0.0011	3.18%	0.0504	ns

Table S14. Moderation effects on **stomatal aperture (Sto)**

Moderator	τ^2	R^2	QM p-value	Permutest
**Donor gene**	0.0395	14.29%	**0.0016**	ns
**Promoter**	0.0460	0.06%	0.7842	ns
**Recipient host**	0.0254	0.0396	**0.0031**	ns
**Recipient group**	0.0460	0.18%	0.8467	ns
**Medium**	0.0395	14.31%	**0.0031**	ns
**Generation**	0.0455	1.17%	0.3776	ns
**Treatment**	0.0011	3.18%	0.0504	ns

Table S15. Moderation effects on **relative electric conductivity (Rec)**

Moderator	τ^2	R^2	QM p-value	Permutest
**Donor gene**	0.0364	0.17%	0.7466	ns
**Promoter**	0.0253	30.72%	0.0609	ns
**Recipient host**	0.0364	0.28%	0.3272	ns
**Recipient group**	0.0364	0.28%	0.3272	ns
**Medium**	0.0364	0.28%	0.3272	ns
**Generation**	0.0365	0.08%	0.5751	ns
**Treatment**	0.0344	5.70%	0.4314	ns

Table S16. Moderation effects on **survival (Sur)**

Moderator	τ^2	R^2	QM p-value	Permutest
**Donor gene**	0.0068	61.77%	**0.0098**	ns
**Promoter**	0.0068	61.77%	**0.0098**	ns
**Recipient host**	0.0068	61.77%	**0.0098**	ns
**Recipient group**	0.0068	61.77%	**0.0098**	ns
**Medium**	0.0177	0.02%	**0.0219**	ns
**Generation**	0.0056	68.53%	**0.0033**	ns
**Treatment**	0.0033	81.63%	**< .0001**	ns

NO_STRESS

Table S17. Moderation effects on **proline** in absence of stress

Moderator	τ^2	R^2	QM p-value	Permutest
**Donor gene**	0.0128	40.79%	0.0601	ns
**Promoter**	0.0160	26.07%	0.1421	ns
**Recipient host**	0.0131	39.64%	**0.0316**	ns
**Recipient group**	0.0216	0.30%	0.9826	ns
**Medium**	0.0208	4.06%	**0.0832**	ns
**Generation**	0.0202	6.52%	**0.0319**	ns

Table S18. Moderation effects on **plant height** in absence of stress

MODERATOR	τ^2	R^2	QM p-value	Permutest
**Donor gene**	0.0011	57.53%	0.1491	ns
**Promoter**	0.0014	47.61%	0.4014	ns
**Recipient host**	0.0009	67.76%	0.0285	ns
**Recipient group**	0.0000	67.76%	0.0285	ns
**Medium**	0.0014	47.61%	0.4014	ns
**Generation**	0.0012	56.08%	0.4066	ns

Table S19. Moderation effects on **seed number** in absence of stress

MODERATOR	τ^2	R^2	QM p-value	Permutest
**Donor gene**	0.0048	24.99%	0.0629	ns
**Promoter**	0.0006	90.55%	**0.0001**	ns
**Recipient host**	0.0048	24.78%	0.4963	ns
**Recipient group**	0.0059	7.30%	0.7818	ns
**Medium**	0.0006	90.55%	**0.0006**	ns
**Generation**	0.0058	8.62%	0.7898	ns

Table S20. Moderation effects on **seed weight** in absence of stress

MODERATOR	τ^2	R^2	QM p-value	Permutest
**Donor gene**	0.0026	33.33%	**0.0184**	ns
**Promoter**	0.0006	84.12%	**0.0362**	ns
**Recipient host**	0.0006	83.06%	**0.0399**	ns
**Recipient group**	0.0006	83.06%	**0.0399**	ns
**Medium**	0.0006	83.06%	**0.0399**	ns
**Generation**	0.0002	94.32%	**0.0676**	ns

Table S21. Moderation effects on **chlorophyll** in absence of stress

MODERATOR	τ^2	R^2	QM p-value	Permutest
**Donor gene**	0.0012	0.66%	0.5480	ns
**Promoter**	0.0001	87.26%	**0.0014**	*
**Recipient host**	0.0001	90.48%	**0.0108**	ns
**Recipient group**	0.0003	78.36%	**0.0067**	ns
**Medium**	0.0003	75.90%	**0.0023**	ns
**Generation**	0.0011	4.15%	0.7667	ns

Table S22. Moderation effects on **root length** in absence of stress

MODERATOR	τ^2	R^2	QM p-value	Permutest
**Donor gene**	0.0048	24.99%	0.0629	ns
**Promoter**	0.0006	90.55%	**0.0001**	ns
**Recipient host**	0.0048	24.78%	0.4963	ns
**Recipient group**	0.0059	7.30%	0.7818	ns
**Medium**	0.0006	90.55%	**0.0006**	ns
**Generation**	0.0058	8.62%	0.7898	ns

Table S23. Moderation effects on **plant weight** in absence of stress

MODERATOR	τ^2	R^2	QM p-value	Permutest
**Donor gene**	0.0000	100.00%	**< .0001**	ns
**Promoter**	0.0001	83.26%	**< .0001**	ns
**Recipient host**	0.0000	100.00%	**< .0001**	ns
**Recipient group**	0.0001	83.30%	**< .0001**	ns
**Medium**	0.0005	0.03%	0.7369	ns
**Generation**	0.0004	21.06%	0.2482	ns

Table S24. Moderation effects on **superoxide dimutase (SOD)** in absence of stress

MODERATOR	τ^2	R^2	QM p-value	Permutest
**Donor gene**	0.0000	100.00%	**< .0001**	ns
**Promoter**	0.0064	49.67%	**0.0283**	ns
**Recipient host**	0.0035	72.76%	**0.0099**	ns
**Recipient group**	0.0064	49.42%	**0.0165**	ns
**Medium**	0.0074	41.97%	0.0958	ns
**Generation**	0.0116	8.78%	0.8608	ns

Table S25. Moderation effects on **malondialdehyde (MDA)** in absence of stress

MODERATOR	τ^2	R^2	QM p-value	Permutest
**Donor gene**	0.0005	91.34%	**< .0001**	ns
**Promoter**	0.0002	96.06%	**< .0001**	*
**Recipient host**	0.0004	93.03%	**< .0001**	ns
**Recipient group**	0.0006	89.87%	**< .0001**	ns
**Medium**	0.0020	63.74%	**0.0274**	ns
**Generation**	0.0017	69.62%	0.0770	ns

Table S26. Moderation effects on **catalase activity (CAT)** in absence of stress

MODERATOR	τ^2	R^2	QM p-value	Permutest
**Donor gene**	0.0200	49.43%	0.0698	ns
**Recipient host**	0.0200	49.43%	0.0698	ns
**Recipient group**	0.0369	6.93%	0.8160	ns
**Medium**	0.0200	49.43%	0.0698	ns
**Generation**	0.0369	6.93%	0.8160	ns

Table S27. Moderation effects on **ascorbate peroxidase activity (APX)** in absence of stress

MODERATOR	τ^2	R^2	QM p-value	Permutest
**Donor gene**	0.0430	0.76%	0.8601	ns
**Promoter**	0.0032	92.56%	**< .0001**	**ns**
**Recipient host**	0.0430	0.76%	0.8601	ns
**Recipient group**	0.0346	20.31%	0.3568	ns
**Medium**	0.0109	74.79%	**0.0193**	ns
**Generation**	0.0300	30.88%	0.4307	ns

Table S28. Moderation effects on **stomatal conductance** in absence of stress

MODERATOR	τ^2	R^2	QM p-value	Permutest
**Donor gene**	0.0003	90.64%	**0.0195**	ns
**Promoter**	0.0016	50.96%	0.0879	ns
**Recipient host**	0.0020	37.38%	0.2045	ns
**Recipient group**	0.0020	37.38%	0.2045	ns
**Generation**	0.0016	50.00%	0.1653	ns

Intercept-present model to evaluate the overall effect size for every moderator category. Tau squared estimated with the Hunter-Schmidt estimator
